# A computational model of PKD and CERT interactions at the trans-Golgi network of mammalian cells

**DOI:** 10.1186/s12918-015-0147-1

**Published:** 2015-02-26

**Authors:** Patrick Weber, Mariana Hornjik, Monilola A Olayioye, Angelika Hausser, Nicole E Radde

**Affiliations:** Institute for Systems Theory and Automatic Control, University of Stuttgart, Pfaffenwaldring 9, Stuttgart, 70569 Germany; Institute of Cell Biology and Immunology, University of Stuttgart, Stuttgart, 70569 Germany

**Keywords:** Computational model, PKD, CERT, Bayesian inference, Trans-golgi network

## Abstract

**Background:**

In mammalian cells protein-lipid interactions at the trans-Golgi network (TGN) determine the formation of vesicles, which transfer secretory proteins to the cellular membrane. This process is regulated by a complex molecular network including protein kinase D (PKD), which is directly involved in the fission of transport vesicles, and its interaction with the ceramide transfer protein CERT that transports ceramide from the endoplasmic reticulum to the TGN.

**Results:**

Here we present a novel quantitative kinetic model for the interactions of the key players PKD, phosphatidylinositol 4-kinase III beta (PI4KIII *β*) and CERT at the TGN membranes. We use sampling-based Bayesian analysis and perturbation experiments for model calibration and validation.

**Conclusions:**

Our quantitative predictions of absolute molecular concentrations and reaction fluxes have major biological implications: Model comparison provides evidence that PKD and CERT interact in a cooperative manner to regulate ceramide transfer. Furthermore, we identify active PKD to be the dominant regulator of the network, especially of CERT-mediated ceramide transfer.

**Electronic supplementary material:**

The online version of this article (doi:10.1186/s12918-015-0147-1) contains supplementary material, which is available to authorized users.

## Background

All transmembrane proteins and soluble proteins that are secreted from a cell into the extracellular space generally pass through the endoplasmic reticulum (ER) and Golgi complex (GC). In mammalian cells the GC constitutes a highly ordered organelle composed of cisternal stacks, the main function of which is the modification of proteins and sorting to their distinct membrane localizations [1]. Cargo is received from the ER at the cis-Golgi complex, traverses the medial Golgi stacks, then reaches the trans-Golgi side where vesicles bud off from the trans-Golgi network (TGN) [2,3]. The formation of these vesicles is driven by a complex interaction of proteins and lipids and is still only partly understood. One of the key players of this network is protein kinase D, which comprises a family of three closely related serine-threonine kinases (PKD1, PKD2 and PKD3). PKD has an established role in the regulation of constitutive but also regulated vesicular transport processes at the TGN [4].

**Figure 1:**
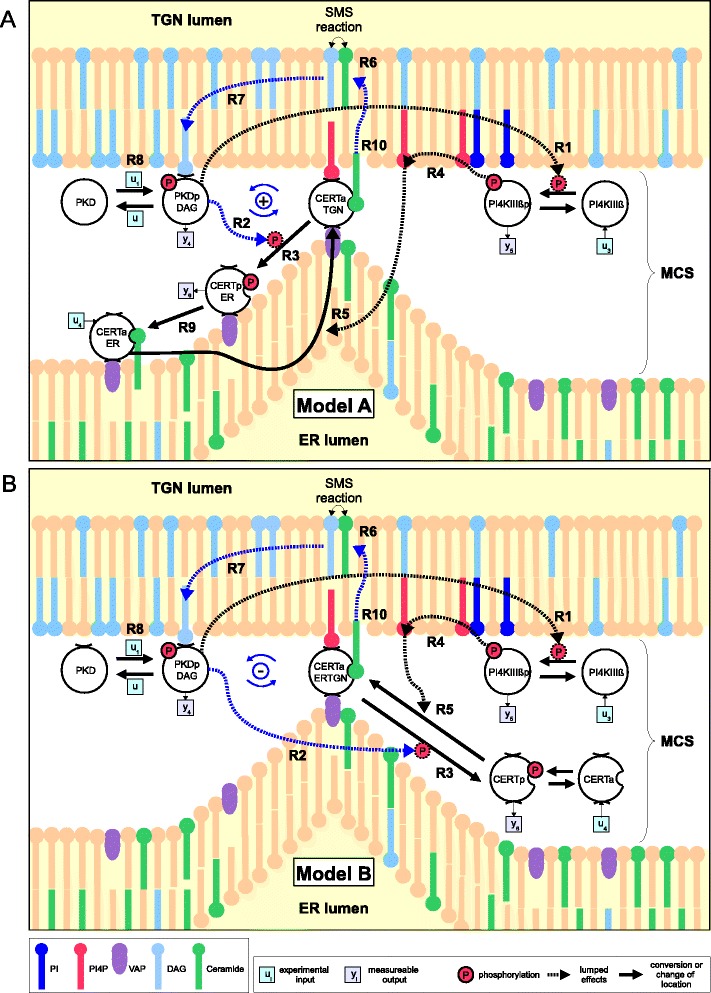
**Model of molecular interactions at the TGN, comprising the key proteins PKD, PI4KIII** ***β*** **and CERT.** Chemical conversions and changes of locations are represented by solid edges with number labels. Catalytic reactions and effective regulatory influences are represented by dashed edges. PKD and PI4KIII *β* are present as inactive or active TGN bound forms (variables PKD, PKDpDAG, PI4KIII *β* and PI4KIII *β*p, respectively). Active TGN bound PKD activates PI4KIII *β* (R1) and detaches CERT from the TGN membrane (R2) via phosphorylation (R3). Active PI4KIII *β* attracts CERT to the TGN (R5) by producing PI4P (R4). Two alternatives for CERT dependent ceramide transport are depicted. **(A)** In model A, ceramide transfer is ensured by a circular reaction scheme of CERT: Unphosphorylated CERT that is bound to both membranes (CERTaTGN) can deliver ceramide to the TGN (R10). Extraction of new ceramide requires detachment from the TGN (R3) via phosphorylation (CERTpER) and subsequent dephosphorylation (R9) at the ER (CERTaER). Thereby, PKD induced CERT phosphorylation (R2) and ceramide transfer (R10) dependent PKD activation (R8) constitute a positive feedback mechanism (R6, R7, R8, R2, R10), indicated by blue arrows. **(B)** In model B, unphosphorylated CERT (CERTa) is only transport-active in an ER-TGN double bound state (CERTaERTGN), constantly transferring ceramide (R10). Here, CERT phosphorylation (CERTp) (R3) causes an interruption of the transfer process and forms a negative feedback between PKD and CERT (R6, R7, R8, R2, R10), indicated by blue arrows.

In the following, we shortly describe the molecular interaction network of PKD at the TGN (Figure 1 and reviewed in [5,6]). The first TGN-localized PKD substrate identified was PI4KIII *β* whose phosphorylation on serine 294 increased the activity of the lipid kinase, resulting in the enhanced production of its substrate PI4P [7]. This phosphorylated lipid is especially important in defining Golgi membrane identity and providing a local signaling platform to which several PI4P-binding proteins are recruited, including the ceramide transfer protein CERT [8,9].

Ceramide is produced at the ER and then transported to the TGN in a non-vesicular manner by CERT, which is recruited by the ER-resident membrane protein VAP [10-12]. The mechanistic understanding is that CERT takes up a ceramide molecule into its START domain, which forms a hydrophobic pocket, followed by the release of ceramide at the TGN membrane [13]. This transport is thought to occur at so-called membrane contact sites (MCS) where the ER and the TGN come into close contact [12]. At the TGN, sphingomyelin synthases (SMS) catalyze the conversion of ceramide and phosphatidylcholine (PC) to sphingomyelin (SM) and diacylglycerol (DAG) [14-16]. DAG fulfills several central functions at the TGN by activating novel PKC isoforms which phosphorylate and activate PKD, by recruiting and activating PKD, and by directly impacting membrane curvature involved in the process of vesicle formation [3,17-19]. CERT, in turn, is phosphorylated by PKD at serine 132 [20], which serves as a priming site for multiple phosphorylations by CKI *γ*2 in the serine rich (SR) motif [21]. This hyperphosphorylation of CERT negatively affects its affinity for PI4P and induces a conformational change that inhibits START domain function [22]. At the ER, CERT is dephosphorylated by PP2C *ε* [23], enhancing the interaction of CERT with the Golgi membranes and also with VAP [22]. Thereby, the proteins PKD, PI4KIII *β* and CERT are involved in a complex scenario of interrelated feedback loops [20]. Despite the knowledge on the qualitative effects of phosphorylation on CERT membrane binding and function, it is currently still unclear how this complex molecular interaction network impacts the efficacy of ceramide transport at ER-Golgi MCS.

Only few studies have attempted to address aspects of TGN function by mathematical modeling. The existing models describe mechanisms for vesicle kinetics, membrane physics, and feedback in the SMS reaction [24-27]. Thus far, no quantitative model exists that describes the kinetics of key protein interactions at the TGN important for secretory transport. Such a quantitative dynamic model would contribute to an increased understanding of molecule interactions and organelle function and is therefore valuable to basic research. In addition, such a model has broader relevance, as the vesicular transport from the TGN to the plasma membrane drives the polarized migration of cancer cells. A further application are biotechnological interventions in the secretory pathway targeting the optimization of the production of therapeutic proteins in mammalian cells [28].

Here, we use mathematical modeling and perturbation experiments to address how the feedback loops within the PKD-CERT network interact to coordinate ceramide transport. We present a quantitative dynamical model for the molecular interactions at the TGN that is based on chemical reaction kinetics. Biochemical time series experiments after perturbation of the system and absolute protein quantification measurements are used for model calibration. As the information in the data is not sufficient to identify all model parameters uniquely, we use statistical Bayesian approaches, which are computationally demanding but particularly tailored to deal with this problem [29-31].

Our calibrated model is able to capture dynamic interactions between PKD, PI4KIII *β* and CERT on an average cellular level. Furthermore, validation experiments confirm that we are able to reliably predict different perturbation scenarios. Our model-based analysis thus provides insight into the regulatory network of key components underlying ceramide transfer at ER-Golgi MCS.

## Methods

### Plasmids and reagents

Expression plasmids encoding Flag-tagged CERT as well as EGFP-tagged CERT, PI4KIII *β*, and PKD2 are described elsewhere [7,20]. The antibodies specific for PKD1 autophosphorylation at serine 910, phosphorylated serine 294 in PI4KIII *β* and phosphorylated serine 132 in CERT have been described elsewhere [7,32,33]. Commercially available antibodies used were as follows: anti-GFP mouse monoclonal (Roche Diagnostics), anti-PI4KIII *β* rabbit polyclonal (Millipore), anti-CERT rabbit polyclonal antibody (Bethyl Laboratories), anti-PKD2 rabbit polyclonal antibody (Cell Signaling), anti-Flag M2 mouse monoclonal antibody (Sigma-Aldrich), anti- *α*-tubulin mouse monoclonal antibody (Millipore). Recombinant purified GFP and GST-PI4KIII *β* were obtained from Vector Laboratories and Life technologies, respectively.

### Cell culture

HEK293T cells were maintained in RPMI 1640 medium supplemented with 10% FCS. For transient transfections, 3·10^5^ HEK293T cells were seeded per well of a 6-well plate. The next day, cells were transfected with 2 *μ*g of plasmid DNA using TransIT293 (Mirus Bio Corporation, Madison, WI) according to the manufacturer’s instructions and grown for 24 hours. Treatment of cells with kbNB142-70 (Tocris) and PDBu (Sigma-Aldrich) was at 10 *μ*M and 1 *μ*M, respectively.

### Protein extraction of cells

Whole cell extracts were obtained by solubilizing cells in lysis buffer (20 mM Tris pH 7.4, 150 mM NaCl, 1% Triton X-100, 1 mM EDTA, 1 mM ethylene glycol tetraacetic acid (EGTA), plus Complete protease inhibitors and PhosSTOP (Roche Diagnostics)). Lysates were clarified by centrifugation at 16,000*g* for 15 min.

### Western blotting

Proteins were separated on a precast 4-12% Bis-Tris polyacrylamide gel (Life Technologies) and blotted onto nitrocellulose membranes (Pall, Dreieich, Germany). After blocking with 0.5% blocking reagent (Roche Diagnostics), filters were probed with specific antibodies. Proteins were visualized with IRdye-coupled secondary antibodies on a Odyssey scanner followed by analysis with Odyssey software (LI-COR Biosciences).

### Image data quantification

Blots have been scanned with the LI-COR ODYSSEE Infrared imaging system. Chanel gain was set to intensity value 5, software gain was varied from 3 to 8 depending on the antibody in use. Gain information is contained on all attached image files. The ’*.tif’ image data was exported with the LI-COR software. For quantification a MATLAB 2011b script and the software’s image analysis toolbox has been used. The script follows recent findings of densitometry research [34] like individual lane background correction and a unified sample tool geometry of one third of average signal width. The image analysis script can be supplied by the authors upon request.

### Software

All calculations are performed using MATLAB R2011b scripts. Models are implemented using MATLAB toolboxes SBtoolbox2 and SBPD package [35]. The ODE solvers embedded in the toolboxes are adopted from the SUNDIALS suite [36]. Relative and absolute solver accuracy has been set to 10^−8^. All MATLAB scripts are available from the authors upon request.

### Bayesian inference strategy

Here we give a short overview of the implemented Bayesian inference strategy, for a more theoretical introduction in ODE modeling and Bayesian inference we refer to Section Background in the supplementary text see Additional file 1. We first perform several repeated minimizations using a bounded local minimization strategy with uniformly sampled initial conditions to gain a set of maximum a posteriori parameters estimates on a logarithmic scale. We then create a log uniform prior distribution for the Bayesian inference centered several orders of magnitude around the best found values (see Additional file 1: Section 1.2). Three repeated runs of a customized version of the parallel tempering MCMC algorithm presented in [37] are performed using ten temperature levels. In each run half a million samples are drawn from the posterior distribution for all temperatures, using MCMC starting values drawn from the best found parameter values from the initial optimizations. Negative results for non-convergence are successfully tested for all sampling runs based on intra-inter-chain variance [38]. A representative parameter subsample (Additional file 2) is used to generate the model predictions. Validation data has not been used for model calibration. Bayes Factors for model comparisons (and their uncertainties) have been calculated for each of the three independent runs via thermodynamic integration [37].

## Results

### Data-driven modeling of PKD, PI4KIII *β* and CERT at the TGN

The exact mechanism by which CERT transfers ceramide from the ER to the TGN is currently still unclear [6,10,13]. In particular, two models have been discussed (see e.g. Figure six in [6]). The ’short distance shuttle’ model suggests that CERT travels 10 nm or more through the cytosol, shuttling between ER and TGN membranes during the ceramide transfer process [11]. By contrast, the ’neck-swinging’ model suggests that CERT is simultaneously bound to both membranes, while extracting ceramide from the ER membranes via its START domain and releasing it at the TGN membrane [2,39]. There are several regulatory differences underlying these distinct transport mechanisms, which we considered by building two different models, A and B: First, PKD-mediated phosphorylation affects CERT transport activity differently in both models. While it has a positive effect on CERT transport activity in the shuttle model, it decreases transport activity in the swinging neck model. Together with an overall positive effect of CERT transport on PKD activity, this constitutes a positive feedback loop for model A and a negative feedback loop for model B, respectively, as illustrated in Figures 1A and 1B. Second, CERT is regulated in a cyclic reaction scheme in the shuttle model, driven by active PKD and active PI4KIII *β*. By contrast, in model B, CERT transfer activity is switched on and off by PI4KIII *β* and PKD, respectively.

Figure 1 shows a schematic scheme of the differences between model A (top) and model B (bottom). Solid lines represent conversions of molecules or changes in localization, whereas dashed lines refer to regulatory or catalytic influences. For the sake of simplicity, some regulations are represented as effective direct regulations which summarize several intermediate reactions.

The figure also shows molecular targets of the perturbation experiments that are used for model calibration, denoted by inputs *u* (ectopic expression of PI4KIII *β* (*u*_3_) and CERT (*u*_4_)), as well as activation and inhibition of PKD (*u*_1_ and *u*_2_, respectively). Moreover, measurable outputs *y* are relative phosphorylation states of PKD (*y*_4_), CERT (*y*_6_) and PI4KIII *β* (*y*_5_).

In both models, active TGN-bound PKD (PKDpDAG) phosphorylates and activates PI4KIII *β* (R1) and, by direct phosphorylation (R2), triggers the detachment of CERT from TGN membranes (R3). At the same time, active PI4KIII *β* produces PI4P (R4), recruiting CERT back to the TGN (R5). In turn, CERT mediated ceramide transfer has an activating influence upon PKD, which comprises SMS driven conversion of PC and ceramide into SM and DAG (R6) and subsequent DAG-mediated recruitment (R7) and activation (R8) of PKD.

In model A (Figure 1A), CERT is permanently associated with the ER via interaction with VAP-A. After delivery of a ceramide molecule to the TGN, PKD-mediated phosphorylation of CERT leads to dissociation from the TGN (R3) while association with the ER remains. Once dephosphorylated at the ER (R9), CERT can deliver a new ceramide molecule to the TGN (R10).

In model B (Figure 1B), the PKD-mediated phosphorylation of CERT leads to its detachment from both the TGN and ER membranes (R3). Thus, only the unphosphorylated CERT molecule is associated with the TGN and the ER and therefore transfer-active. Dephosphorylation of CERT is required for MCS re-recruitment.

Based on the current state of knowledge about these molecular interactions and on available measurements, we used chemical reaction kinetics to build differential equation models for the two model variants, which resulted in systems with seven state variables and 26 (model A) and 27 (model B) parameters, respectively. Model equations and details are given in the Additional file 1: Section 1.3 and Table S1.

### Absolute measurements for model calibration

**Figure 2:**
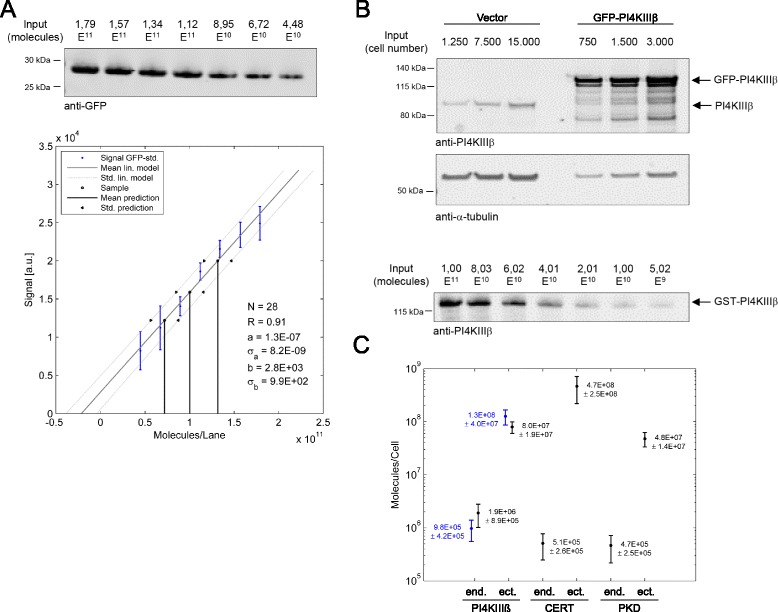
**Absolute quantification of endogenous proteins in HEK293T cells.** **(A)** Top: Different amounts of recombinant GFP were loaded on a polyacrylamide gel and visualized by Western blot analysis using a GFP-specific antibody. Bottom: Linear regression model analysis of the recombinant GFP signals. **(B)** Top: HEK293T cells were transfected with an empty vector as a control or a plasmid encoding GFP-tagged PI4KIII *β* and cultured for 24 h. Cells were lysed, and expression of endogenous and ectopically expressed PI4KIII *β* was detected by Western blot analysis using a PI4KIII *β*-specific antibody. Detection of tubulin served as a loading control. Bottom: Different amounts of recombinant GST-tagged PI4KIII *β* were loaded on a polyacrylamide gel and visualized by Western blot analysis using a PI4KIII *β*-specific antibody. **(C)** Comparison of absolute quantifications via GST-PI4KIII *β* standard (blue) and GFP-standard (black).

Calculation of absolute protein amounts per cell is a prerequisite to establish a quantitative model [40]. To do so, we used an indirect method making use of a commercially available recombinant GFP standard. Decreasing numbers of recombinant GFP molecules were detected by Western Blot analysis using a GFP-specific antibody (Figure 2A, top). The band intensity was quantified to generate a standard curve connecting signal strength and protein amount (Figure 2A, bottom). Next, we transiently transfected HEK293T cells with a plasmid encoding GFP-tagged PI4KIII *β*. Cells were lysed 24 hours later and expression of GFP-tagged PI4KIII *β* was detected by quantitative Western Blot analysis using the GFP-specific antibody. In parallel, cell lysates were probed with a PI4KIII *β*-specific antibody, detecting endogenous and ectopically expressed PI4KIII *β* at the same time (Figure 2B, top). Based on the GFP signal, we determined the amount of GFP-PI4KIII *β* expressed in a defined number of cells by inverse regression using the GFP standard curve (for details see Section 2 in the Additional file 1). Subsequently, we calculated the absolute amount of endogenous PI4KIII *β*, which accounted to approximately two million molecules per cell (Figure 2C) by determining the signal ratio between endogenous and ectopically expressed protein from Figure 2B. We then validated this result with a commercially available purified recombinant GST-PI4KIII *β* protein. Similarly to the GFP standard, we detected decreasing amounts of GST-PI4KIII *β* by Western Blot analysis using a PI4KIII *β*-specific antibody (Figure 2B, bottom) and correlated the signal intensity with the number of molecules. Indeed, the results were in the same order of magnitude and in good agreement with respect to the error estimates of both methods thus confirming our calculation (Figure 2C). Likewise, we calculated the amount of endogenous and ectopically expressed PKD and CERT and integrated these absolute estimates together with the estimated standard deviation into our dataset. Our results show that a single cell contains roughly half a million PKD and CERT molecules each. The numbers of the ectopically expressed proteins ranged from ten (GFP-tagged PKD1) to hundred million (GFP-tagged CERT) molecules per cell (Figure 2C). Coefficients of variation are determined to be between 25 to 40%, accurate enough to estimate the order of magnitude of a protein.

### Time series measurements for model calibration

**Figure 3:**
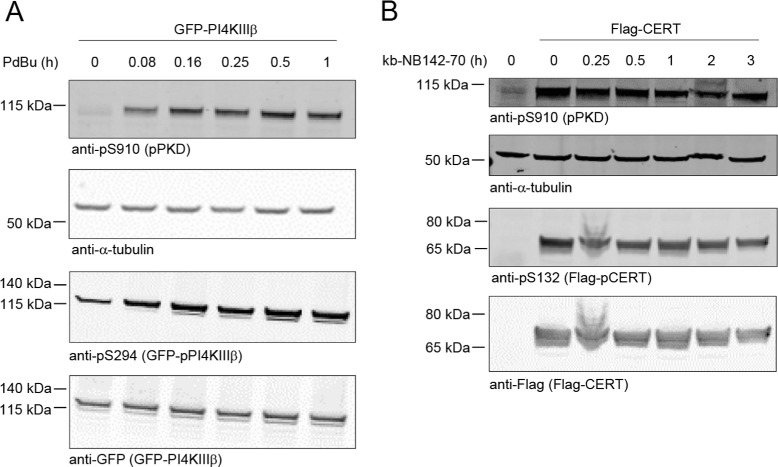
**Time series experiments.** **(A)** HEK293T cells were transfected with a plasmid encoding GFP-tagged PI4KIII *β* and cultured for 24 h. Cells were stimulated with PDBu for the indicated time points, lysed, and phosphorylation and expression of PI4KIII *β* were analyzed by Western blot analysis using a pS294- and a GFP-specific antibody, respectively. Autophosphorylation of endogenous PKD was detected using the pS910-specific antibody. Detection of tubulin served as a loading control. Shown is a representative experiment, n = 3. **(B)** HEK293T cells were transfected with a control vector or a plasmid encoding Flag-tagged CERT and cultured for 24 h. Control cells were left untreated, whereas Flag-CERT transfected cells were treated with the PKD selective inhibitor kbNB142-70 for the indicated time points. Afterwards cells were lysed, and phosphorylation and expression of CERT were analyzed by Western blot analysis using a pS132- and a Flag-specific antibody, respectively. Autophosphorylation of endogenous PKD was detected using the pS910-specific antibody. Detection of tubulin served as a loading control. Shown is a representative experiment, n = 3.

In order to identify perturbations leading to a significant system response, we performed experiments in which we detected dynamic changes of the phosphorylation of PKD and its substrates PI4KIII *β* and CERT. Since the antibodies specific for phosphorylated CERT (pS132) and PI4KIII *β* (pS294) are not suited for the detection of the endogenous proteins we ectopically expressed CERT and PI4KIII *β* while additionally perturbing endogenous PKD activity by adding a respective activator and inhibitor to the cell cultures. First, we ectopically expressed GFP-tagged PI4KIII *β* in HEK293T cells for 24 hours and subsequently left cells untreated or stimulated the cells with Phorbol 12,13-dibutyrate (PDBu), a potent activator of PKD [41], see Figure 3A. Cells were harvested at different time points, and the level of active PKD was analyzed using an antibody specific for autophosphorylated PKD (pS910). In parallel, we detected the expression and the PKD-mediated phosphorylation of GFP-PI4KIII *β* using the GFP-specific and pS294-specific antibodies, respectively. Stimulation of cells with PDBu steadily increased PKD activity within one hour. Likewise, PKD-mediated phosphorylation of PI4KIII *β* was strongly enhanced during this time.

In a second experiment, we ectopically expressed Flag-tagged CERT, see Figure 3B. After 24 hours, cells were left untreated or treated with the selective PKD inhibitor kb-NB142-70 [42] for the indicated time. Activation of PKD as well as phosphorylation and expression of Flag-CERT were analyzed using pS910-specific and pS132 and Flag-specific antibodies, respectively. Expression of Flag-CERT strongly increased endogenous PKD activity, as can be seen from comparison of the ’0’-lanes in Figure 3 B. This activation, however, was decreased upon treatment of cells with kb-NB142-70. The strongest reduction in PKD activation was detected after treating cells with kb-NB142-70 for 3 hours. Likewise, PKD-mediated basal phosphorylation of CERT was reduced in kb-NB142-70 treated cells compared to untreated cells, see Additional file 1: Section 3.1 and 3.2.

### Bayesian parameter estimation

Both datasets (Additional file 3), absolute protein amounts and relative phosphorylation states after the perturbations described were used for model calibration in a statistical Bayesian framework. Therefore, we interpreted the measurements as random variates that were generated by a stochastic process, which is defined as the deterministic solution of the differential equation model perturbed by additive stochastic noise terms. The resulting stochastic model allows us to define the likelihood function, which assigns for each set of parameters *θ* the probability *p*(*y*|*θ*) to obtain the dataset *y*. Especially in a Bayesian approach parameters are interpreted as random variables as well. Prior knowledge about these are included via prior distributions *p*(*θ*) in the parameter space, which reflect the state of knowledge about the parameters before having seen the data. The objective in a Bayesian approach is the posterior distribution *p*(*θ*|*y*), the conditional distribution in the parameter space after having taken the data into account, which is according to Bayes’ Theorem proportional to the product of the prior distribution and the likelihood function, see Additional file 1: Section 1.1 and 1.2.

**Figure 4:**
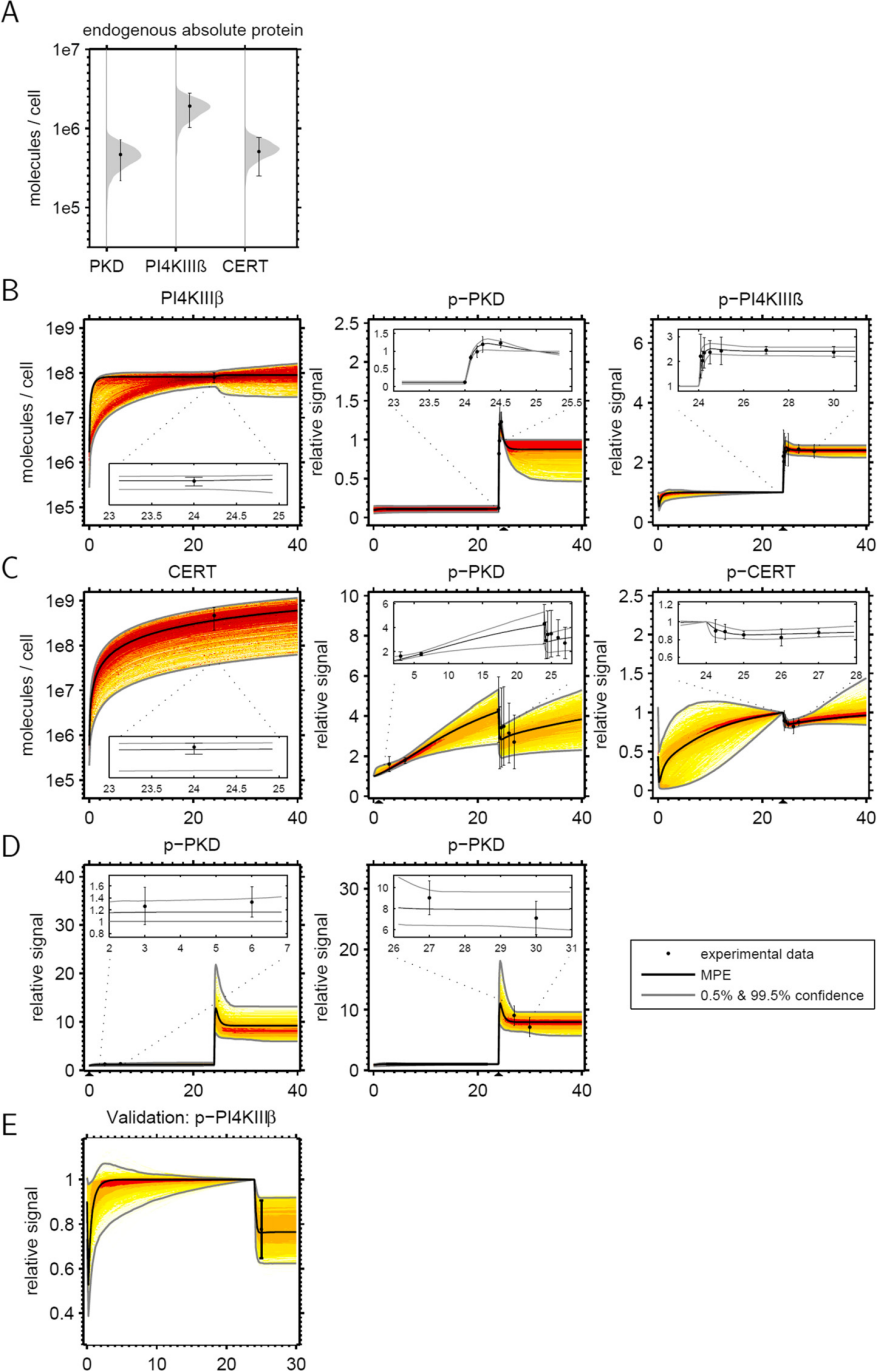
**Calibration of model A to experimental data.** **(A)** Endogenous concentrations of PKD, PI4KIII *β* and CERT from the absolute quantification experiments are depicted together with the model fit of the steady state distributions. **(B)** Model fit of ectopic expression of PI4KIII *β* with subsequent activation of PKD via PDBu at 24 h. Depicted are the the rise of the overall PI4KIII *β* level and the change of PKD and PI4KIII *β* phosphorylation after PDBu addition. **(C)** Model fit of ectopic expression of CERT with subsequent inhibition of PKD via kb-NB142-70 after 24 h. Shown are the the rise of the overall CERT level and the change of PKD and CERT phosphorylation after kb-NB142-70 addition. **(D)** Refinement experiments focusing on measurements of early PKD phosphorylation during ectopic expression of PI4KIII *β* and long time change in PKD phosphorylation up to 6 hours after PDBu addition. **(E)** Validation experiment. PI4KIII *β* phosphorylation was measured at 25.25 h after ectopic expression of PI4KIII *β* and inhibition of PKD via kb-NB142-70 at 24 h. Data were not used for model calibration.

We investigated this distribution by generating samples via Markov Chain Monte Carlo sampling (Additional file 1: Section 4 and 5). These samples were subsequently used to estimate distributions for various quantities via Monte Carlo integration. Figure 4 shows a comparison of the calibrated model A with the set of experimental data that was used for calibration. Absolute endogenous protein amounts and respective distributions predicted by the model are shown in Figure 4A. As can be seen, both agree very well for all three proteins.

The data shown in Figures 4B-E correspond to time series measurements after system perturbations. Experimental data are represented as mean with standard deviations, while respective model predictions represent time-varying densities for the states of the system, which are illustrated by different colors (density is increasing from yellow to dark red). The maximum a posteriori estimate that maximizes the posterior distribution is depicted as solid black line. Outer lines represent the 99.5% confidence intervals estimated from the sample.

The data shown in Figure 4B correspond to the perturbation experiment in Figure 3A, *i.e.* ectopic expression of PI4KIII *β* and subsequent activation of PKD with PDBu. The absolute amount of PI4KIII *β* molecules per cell were quantified as described after 24 h vector expression, followed by time series experiments of phospho-PKD and phospho-PI4KIII *β* within a few hours after treatment with PDBu. The model suggests that PI4KIII *β* rapidly reaches a new steady state after a fast increase within 24 hours. Furthermore, PKD activity rapidly rises after treatment with PDBu, followed by PI4KIII *β*. Both active kinases reach a new steady state approximately 1-2 hours after treatment.

The model fit to the perturbation experiment in Figure 3B, ectopic expression of CERT and subsequent inhibition of PKD, is shown in Figure 4C. In contrast to Figure 4B, in which PI4KIII *β* was ectopically expressed, the model suggests that CERT still increases after 24 hours of vector expression. Inhibition of PKD causes a rapid decrease in phospho-PKD, followed by a decrease in phospho-CERT, which is well reflected in our model. Our simulations suggest that this is a transient decrease due to the continuing increase in the overall CERT amount.

Figure 4D depicts model-based refinement experiments, which were particularly chosen in order to reduce the variance in model predictions, as described in [43]. These are PKD activation early after ectopic expression of PI4KIII *β*, and the long-term behavior of PKD 30 hours after treatment with PDBu.

Overall, Figure 4 shows a very good agreement of experiments and predictions of model A for all scenarios. The figure also shows that the model uncertainty is significantly reduced for time points near experimental data. Moreover, while some uncertainty remains for variables and time points far away from measurement instances, the flexibility of the model was significantly reduced by the data, which led us conclude that the model is not too complex and the data contain considerable information about model parameters.

The respective figures for model B are shown in Additional file 1: Figure S6 and Section 6.2. The data fit of model B shows two major differences. First, model B is not able to reproduce the experimentally observed early increase of PKD activity (Additional file 1: Figure S6D, in comparison to Figure 4). Second, model B suggests an overshoot of phosphorylated CERT at the beginning of the ectopic expression of CERT in Additional file 1: Figure S6C, which was not the case for model A (Figure 4C).

In addition to this visual inspection, we decided to use the Bayesian framework for a sophisticated model comparison that also takes differences in model complexity into account. For this purpose we used thermodynamic integration to calculate the marginal likelihoods of both models, which indicates the expected likelihood to observe the data, averaged over all possible parameterizations. Then we used the Bayes factor *K*_*A*,*B*_, which is the ratio of these two probabilities, to rank the two model variants. The logarithmic Bayes factor reads 2 log*K*_*A*,*B*_=12.3±0.4, with standard deviation that was estimated from three repeated sampling runs. Hence, this analysis renders model A superior to model B with very strong evidence, as described in [44] (see Additional file 1: Sections 4.3, 5 and 6.1).

Summarizing these results, our model study favors model A, in which CERT and PKD regulate CERT transport activity in a cooperative manner. Thus, we decided to focus primarily on the analysis of model A in the following.

Finally, Figure 4E shows an independent validation experiment that was not used for parameter estimation but conducted after the model was used for a prediction of this scenario. Here, PKD activity was inhibited 24 h after ectopically expressing PI4KIII *β* and phospho-PI4KIII *β* was measured 60 min after inhibition (see Additional file 1: Figure S1B). It can be seen that also in this scenario model prediction and experiment are in very good agreement. Thus, our model not only reproduces a set of different experiments, but can also be used for reliable predictions of new perturbation scenarios.

Hence we are equipped with an appropriate model that captures absolute protein levels and dynamic responses of the system to different perturbations.

### The endogenous TGN: highly responsive regulatory couplings

**Figure 5:**
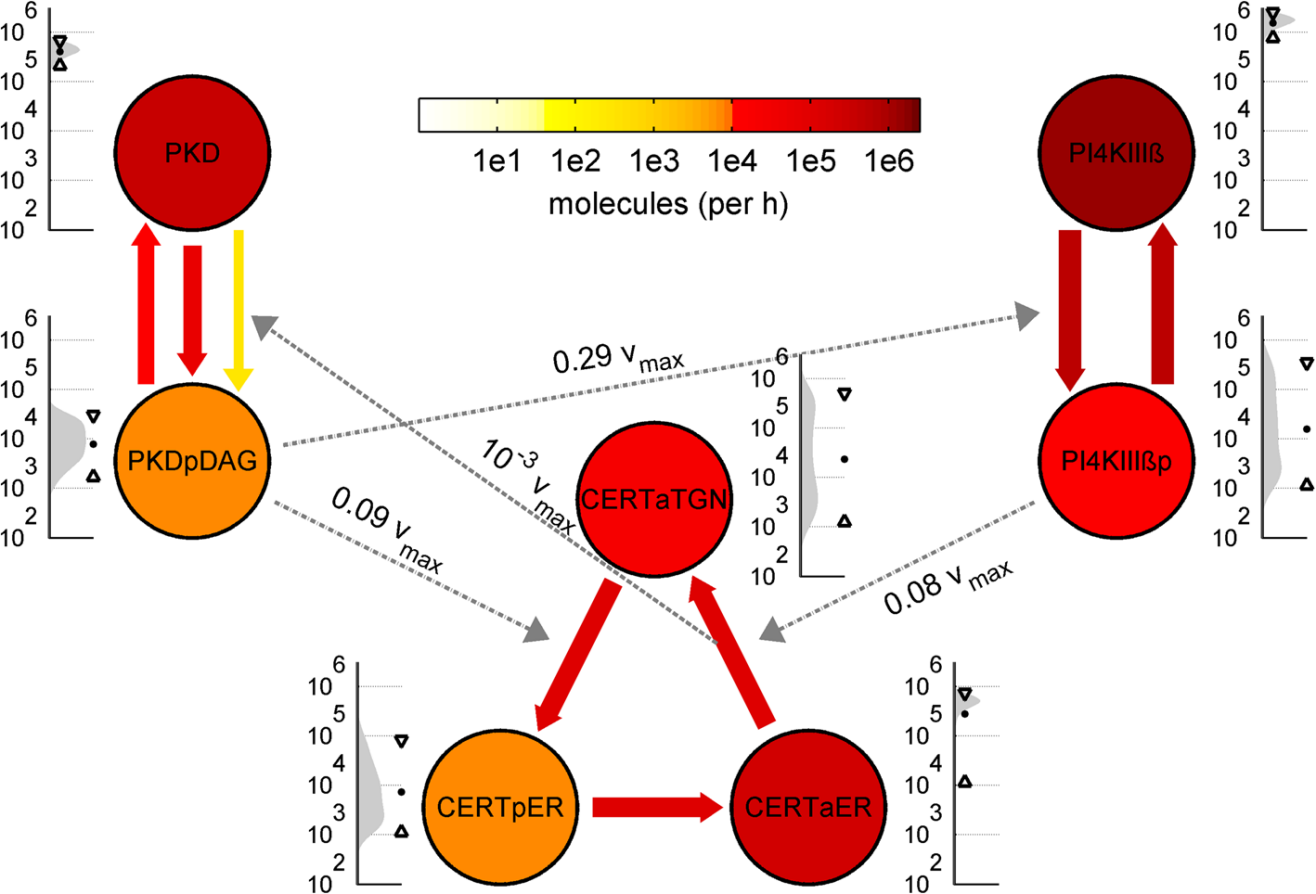
**Prediction of the endogenous TGN.** Prediction of protein amounts, fluxes and regulatory influences of the endogenous TGN. Model A is simulated multiple times using a representative parameter sample from the posterior distribution with all experimental inputs inactive. The simulation represents the endogenous Golgi of an average cell. Units for nodes are molecules per cell and flow rates are given in molecules per hour. Mini diagrams next to the nodes depict the steady state distributions of the endogenous abundances with expectation values and 0.5% and 99.5% confidence intervals on a logarithmic scale. Color encoding of nodes and edges represent expectation values of abundances and flow rates. In the case of PKD there are two activation reactions (basal and CERT dependent). Catalytic influences are represented with dashed arrows and are modeled with Michaelis Menten similar kinetics. Expectation values of the operating points for these regulations are given in units of the respective maximum reaction rate. Values far below *v*_*max*_ depict non-saturated coupling.

We next use model A to predict and analyze the network in the endogenous state. Therefore, we simulated the system without any perturbations by setting all inputs to zero. The system reaches a dynamic equilibrium in which concentrations and fluxes are constant and ceramide is constantly extracted from the ER and released to the TGN membrane. We calculated distributions for the concentration of each molecular species, as well as distributions for fluxes and regulatory influences in the network. The result is illustrated in Figure 5. Expectation values for molecule numbers per cell and fluxes in molecules per hour are visualized by different colors on a logarithmic scale. Additionally, distributions for concentrations are shown next to each node along with expectation values and 99.5% confidence intervals.

In accordance with the experimental absolute quantification experiments (Figure 2), PI4KIII *β* is the most abundant protein with about 1 mio molecules per cell, while PKD and CERT are both present with about half a million molecules. Furthermore, most PKD and PI4KIII *β* molecules are present in their inactive non-phosphorylated state, while there remains large uncertainty for the active states.

Flux rates correlate positively with respective protein abundances. Accordingly, highest fluxes are encountered for phosphorylation and dephosphorylation of PI4KIII *β*, followed by lower rates for PKD and CERT. Basal phosphorylation and dephosphorylation rates of PKD are two orders of magnitude higher than ceramide transfer dependent activation. The model also predicts that the majority of CERT molecules is unphosphorylated. However, the rates of phosphorylation and dephosphosphorylation are rather similar, indicating that dephosphorylation at the ER is very efficient. An uncertainty of several orders of magnitude is predicted for TGN bound CERT (CERTaTGN), suggesting that our data agrees with different TGN resting times.

Regulatory influences that represent the feedback in the network are indicated by gray lines. They have been modeled with Michael Menten like kinetics that are upper bounded with maximal rates *v*_*max*_. Regulation strengths are given in terms of these *v*_*max*_ values. As can be seen in Figure 5, all regulation strengths are far below their saturation values, indicating that they are highly sensitive to changes in molecular concentrations of the regulators.

Summarizing, we are equipped with a quantitative holistic picture of endogenous TGN functioning including also uncertainties. The overall picture suggests that the system operates in a state that is highly responsive to changes in the regulator concentrations.

### Active PKD regulates CERT dependent ceramide transfer

**Figure 6:**
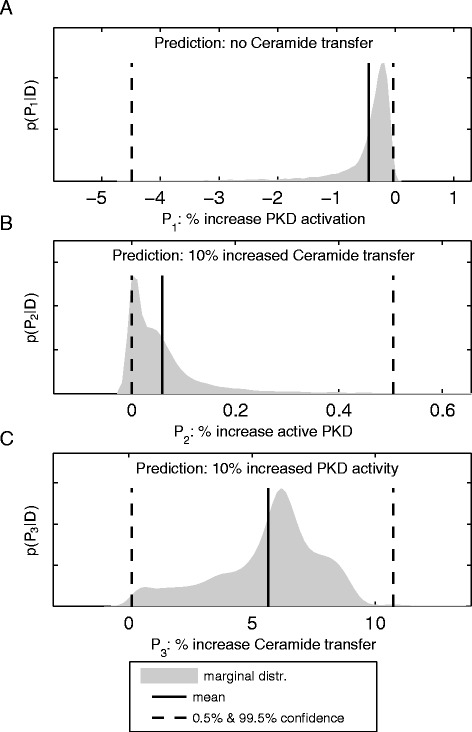
**Model responses to perturbation of PKD and CERT-mediated ceramide transport.** **(A)** The influence of CERT mediated ceramide transfer on PKD activation is set to zero in the model. Expected PKD activation drops by less than 1 percent. **(B)** Ceramide transfer is increased by 10 % in the model. Expected PKD activity increases by 0.1%. **(C)** PKD activity is increased by 10% in the model. Expected ceramide transfer increases by 6%.

The question arises whether this sensitivity of single edges also results in a highly responsive overall network response of the closed loop feedback system and which of the key players determines the operating state of the endogenous system. We therefore analyzed the mutual influence of the phosphorylation state of PKD and CERT transport activity. Simulation results are shown in Figure 6. Figure 6A shows the relative change in PKD activity in the case that ceramide transport activity of CERT is completely switched off. It can be seen that PKD activity is only slightly affected in this scenario, with an expected increase of less than 1% with a rather small variance. The model preserves PKD activity via the basal ceramide independent phosphorylation reaction, see Figure 5. Similarly, PKD activity shows an increase of about only 0.1% in response to an increase in ceramide transfer activity by 10% (Figure 6B). These results indicate that PKD activity is only marginally influenced by CERT transport activity and that CERT has a rather weak effect on PKD activity. By contrast, a 10% increase in PKD activity resulted in an expected increase in CERT transport activity of about 6% (Figure 6C), leading to the conclusion that PKD is a good candidate to regulate CERT transport activity. In summary, these results identify PKD as an important regulator of the systems operating point, which is in turn not much affected by CERT.

## Discussion

In this study we present for the first time a quantitative dynamic model describing the phosphorylation-dependent CERT-mediated regulation of ceramide transfer between ER and Golgi membranes. Our model comprises the complex interrelated feedback loops of interdependent TGN-localized protein and lipid kinases on the activity of the lipid transfer protein CERT. For model calibration we used statistical sampling-based Bayesian inference techniques, which yield beside point estimates also information about uncertainties in terms of probability distributions. Our computational model was able to reproduce the kinetic response of the molecular interaction network comprising PKD, PI4KIII *β* and CERT in terms of phosphorylation states to a set of different perturbation experiments (Figures 3 and 4). Furthermore, our model reliably predicted the behavior to new perturbations, which were experimentally verified, validating the predictive power of the model.

Using Bayesian model comparison techniques, we were able to rank model variants that differ in the impact of phosphorylation of CERT at the SR motif regarding its ceramide transfer activity. In model A (Figure 1), which is supported by our study, PKD-mediated phosphorylation of CERT constitutes an overall positive feedback loop between CERT transfer activity and PKD activity. By contrast, in model B, this feedback has a negative effect on CERT-mediated ceramide transfer. Our results thus suggest that CERT and PKD interact in a cooperative rather than a competitive manner to regulate ceramide transfer activity. An experiment that helped to distinguish between the two models was the ectopic expression of PIKIII *β*, followed by the measurement of the phosphorylation and thus activation state of PKD (Figure 4D). Model A was able to mimic the experimentally observed early increase in PKD phosphorylation, which was not the case for model B. In the latter model this is due to an immediate buffering of this positive effect by the strong negative feedback on CERT mediated ceramide transfer via PKD phosphorylation.

Although energy-consuming, the circular reaction scheme has the advantage that it can rapidly respond to changes at the level of the ER or Golgi to either accelerate or attenuate lipid metabolic pathways required for vesicle formation at the TGN. Indeed, our model predicts a high responsiveness for all regulations in the endogenous system to changes in the regulator concentrations.

Additionally, we predicted protein amounts and their phosphorylation states, as well as fluxes between these states and strengths of regulatory effects within the entire molecular network for the endogenous system (Figure 5). Predicted absolute protein amounts were in good agreement with corresponding measurements (Figure 2). In HEK293T cells, PI4KIII *β* is the most abundant protein with about 1 million molecules per cell, followed by PKD, while the average amount of CERT molecules per cell is about one order of magnitude lower. Moreover, our model predicts that the majority of both the PKD and PI4KIII *β* molecules are present in an inactive unphosphorylated state, while most CERT molecules are unphosphorylated and thus transfer-active. Unfortunately, there is currently limited literature about absolute protein abundances in HEK293T pathways, making a direct comparison of our quantification results difficult. However, a recent study of the Wnt pathway in HEK293T cells reports an average abundance of 1.6·10^5^ copies per cell for these signaling molecules [45]. This is in the same order of magnitude as our average abundance of the key proteins for the endogenous system, which we estimated to be 7·10^5^ copies per cell.

Our simulations furthermore suggest that PKD has a strong regulatory effect on CERT transfer activity, while, in turn, PKD activity is only modestly affected by the amount of ceramide that is delivered to the TGN via CERT, rendering PKD a key regulator of the whole network state (Figure 6). This also suggests that at the TGN, the DAG pool produced as a consequence of CERT-mediated lipid transfer plays a minor role in PKD recruitment and/or activation. Therefore, alternative pathways, such as the hydrolysis of PI(4,5)P _2_ by PLC *β*3 [46] or dephosphorylation of phosphatidic acid by lipid-phosphate phosphatases [26,47] most likely provide DAG to locally regulate PKD at the TGN membranes.

One limitation of our current model is its calibration using data derived from ectopic expression experiments. The predictions made for the behavior of the endogenous system thus contain large uncertainties. However, early PKD responses and absolute protein measurements were obtained in minimally perturbed cells. The model is currently calibrated with data from HEK293T cells since this cell line is well suited for experimental interventions like transfections with expression constructs. However, multiple interactions in our model have also been identified in other mammalian epithelial cell lines like HeLa cells, and structural homology in the regulation of the TGN has been observed [4,18].

An important issue in Bayesian learning approaches is the impact of the prior distribution on the results. Since there was limited quantitative literature data that could directly be included into our model, we decided to work with a rather conservative approach. We used log uniform prior distributions for all parameters, which cover a wide range of orders of magnitude. Bounds were taken from the literature whenever available, such as for example in case of protein half lifes or protein amounts [48,49]. In addition, we took initial parameter optimization runs into account to set sufficiently conservative estimates for upper and lower bounds. Thus our priors are rather uninformative. Since the data contains quite some information about the model, as can be seen by the model fits, we believe that our results are not dominated by the prior distributions. However, as it is usually the case with Bayesian inference, we are aware that the results depend on the parametrization and thus might look different when using uniform priors after a transformation of the parameters.

In the future, our model is not only useful to predict perturbations at the protein level, but can also serve as a starting point to investigate further molecular interactions of interest via suitable model extensions. First, by combining the model developed here with a previous model describing the regulation of the SMS1 reaction at the TGN [27], it will be possible to predict lipid synthesis and transfer rates *in vivo*, which are difficult to measure experimentally.

Second, experimental results described in [22] provide ideas for further extensions regarding CERT transport activity. While our model is qualitatively able to reproduce the observed decrease in ceramide transport of a CERT-10E mutant, which mimics the hyperphosphorylated state, capturing the behavior of the S132A mutant that cannot be phosphorylated at the SRM, would require structural extensions. Furthermore, it was reported that the amount of sphingomyelin in the plasma membrane affects CERT activity via the regulation of its phosphorylation state, thereby constituting a negative feedback mechanism in cellular lipid homeostasis. Interestingly, phosphorylation at an additional site, namely serine 315, via a still unknown kinase was recently reported to enhance the affinity of CERT for VAP at the ER, thereby upregulating ER to Golgi trafficking of ceramide [50]. The post-translational modification of CERT by phosphorylation thus appears to be a central general mechanism by which ceramide transfer at MCS is regulated.

Third, alternative metabolic pathways that contribute to local DAG generation at the TGN membranes can be integrated into our model, enabling the study of the dynamic behavior of PKD activation and/or recruitment [51].

Finally, it will be interesting to integrate into our model additional proteins such as the sterol transfer protein OSBP. Similar to CERT, OSBP contains an FFAT motif and a PH domain which target the protein to ER and Golgi membranes, respectively [13]. Importantly, OSBP is also regulated by PKD-mediated phosphorylation and is thought to stabilize CERT localization at MCS, thereby coordinating sphingomyelin synthesis with sterol metabolism [52].

## Conclusions

In sum, our computational analysis of ceramide transport between the ER and Golgi membranes reveals the cooperativity between PKD and CERT and identifies PKD as the critical regulator in the system. Based on these findings and its further implications, we believe that our quantitative computational model constitutes a valuable contribution towards the holistic mechanistic understanding of the molecular network supporting Golgi secretory function.

## Availability of supporting data

The data sets supporting the results of this article are included within additional files.

## Additional files

Additional file 1
**Supplementary text.** Description: A pdf-file including a detailed theoretical introduction to ODE models, Bayesian Inference and the employed MCMC sampling algorithm. It includes tables of the model equations, further supporting experimental results and explanations for data normalization and error calculus.

Additional file 2
**Model files and parameters.** Software needed: MATLAB R2011b (or higher), SBtoolbox2, SBML viewer, text editor. A compressed archive containing the simulation models A and B (SBML and SBtoolbox2 formats). It includes general and SBtoolbox2 specific simulation instructions (readme.txt, experiment.exp) and two MATLAB Data-files containing double precision variables of 5000 representative parameter samples.

Additional file 3
**Experimental data.** Software needed: spreadsheet viewer (MS Excel, Libre Office, Open Office), Adobe Reader. A compressed archive containing a xls-sheet with readily quantified image data and two pdf-files with a summary of the raw image data in form of expert labeled Western Blots.

## Abbreviations

TGN: trans-Golgi network
ER: Endoplasmic reticulum
MCS: Membrane contact site
PKD: Protein kinase D
PI4KIII *β*: Phosphatidylinositol 4-kinase III beta
CERT: Ceramide transfer protein
PI4P: Phosphatidyl-4-phosphate
SMS: Sphingomyelin synthase
PC: Phosphatidylcholine
SM: Sphingomyelin
DAG: Diacylglycerol
VAP: Vesicle associated membrane associated protein
PP2C *ε*: Protein phosphatase 2C epsilon
CKI *γ*2: Casein kinase I gamma-2
START: Steroidogenic acute regulatory protein
FFAT: Two phenylalanine in an acidic tract
HEK293T: Human embryonic kidney cells 293
PDBu: Phorbol 12,13-dibutyrate
Wnt: Wingless int
PI(4,5)P _2_: Phosphatidylinositol 4,5-bisphosphate
PLC *β*3: Phospholipase C beta 3
OSBP: Oxysterol-binding protein
MCMC: Markov Chain Monte Carlo
