# Supplementary material for: A computational model of PKD and CERT interactions at the trans-Golgi network of mammalian cells
Source: BMC Syst Biol. 2015 Feb 26;9:9. doi: 10.1186/s12918-015-0147-1 (PMC4349302; doi:10.1186/s12918-015-0147-1)
Supplement: Additional file 1 — Supplementary text. Description: A pdf-file including a detailed theoretical introduction to ODE models, Bayesian Inference and the employed MCMC sampling algorithm. It includes tables of the model equations, further supporting experimental results and explanations for data normalization and error calculus. [file 12918_2015_147_MOESM1_ESM.pdf]

Supplement - A computational model of PKD and CERT interactions at the trans-Golgi network of mammalian cells

Patrick Weber[1], Mariana Hornjik[2], Monilola A Olayioye[2] Angelika Hausser[2] Nicole Radde[1,*]
1 Institute for Systems Theory and Automatic Control, University of Stuttgart, Pfaffenwaldring 9, 70569 Stuttgart, Germany
2 Institute of Cell Biology and Immunology, University of Stuttgart, Allmandring 31, 70569 Stuttgart, Germany.
∗ Corresponding Author, E-mail: Nicole.Radde@ist.uni-stuttgart.de

1 Modeling and statistical Bayesian inference

1.1 The likelihood function

Our models are systems of parametrized ordinary differential equations given in state space representation,

$$\Sigma : \begin{cases} \dot{x}(t; u, \theta) = f(x(t; u, \theta), u(t), \theta), & x(0; u, \theta) = x_0(u, \theta), \\ y(t; u, \theta) = h(x(t; u, \theta), u(t), \theta), & \end{cases}$$

with state vector $x(t; u, \theta) \in \mathbb{R}_+^{n_x}$, parameters $\theta \in \mathbb{R}_+^{n_\theta}$, outputs $y(t; u, \theta) \in \mathbb{R}_+^{n_y}$ and inputs $u(t) \in \mathbb{R}_+^{n_u}$. Initial conditions x_0 might depend on inputs and parameters.

For the estimation of parameters with experimental data we have to specify input vectors $u^i(t)$ and a set of measurable outputs

$$Y^i = \{y_j^i(t_k)\}_{j \in \mathcal{I}^i, t_k \in \mathcal{J}_j^i} \text{ with } \mathcal{I}^i \subseteq \{1, \ldots, n_y\},\ \mathcal{J}_j^i \subset \mathbb{R}_0^+$$

for each of the $i = 1 \ldots n_\epsilon$ experiments, which we refer to as experiment description $\mathcal{E}$. Here, index i enumerates different experiments, index j defines the measured outputs, which may vary in each experiment, and k enumerates different time points, which might also differ for different experiments and different outputs.

The data Z is assumed to be generated by a stochastic process defined by

$$z_j^i(t_k) = y_j^i(t_k; u^i, \theta^*) + \eta_{i,j,k} \text{ with } \eta_{i,j,k} \sim \mathcal{N}(0, {\sigma^*}^2_{i,j,k}),$$

which describes the solution of the ODE system with the true parameters θ^*, that is perturbed by independent additive Gaussian noise with variance ${\sigma^*}^2_{i,j,k}$.

We refer to the data of a single experiment as $\mathcal{D}^i = \{z_i^j(t_k)\}_{j \in \mathcal{I}_j, t_k \in \mathcal{J}_j^i}$, while the complete dataset is defined by $\mathcal{D} = \{D^i\}_{i=1}^{n_\mathcal{E}}$.

The likelihood function of this stochastic model reads

$$\begin{aligned} \mathcal{L}_\mathcal{D}(\theta) &= \prod_{i=1}^{n_\mathcal{E}} \mathcal{L}_{\mathcal{D}^i}(\theta) \\ &= \prod_{i=1}^{n_\mathcal{E}} \prod_{j \in \mathcal{I}_j} \prod_{t_k \in \mathcal{J}_j^i} \frac{1}{\sqrt{2\pi}\hat{\sigma}_{i,j,k}} \exp\left\{ -\frac{1}{2} \left(\frac{z_i^j(t_k) - y_i^j(t_k; u^i, \theta)}{\hat{\sigma}_{i,j,k}} \right)^2 \right\}. \end{aligned}$$

For the sake of simplicity, we plug in the unbiased empirical variance estimate $\hat{\sigma}_{i,j,k}$ for $\sigma^*_{i,j,k}$ from experimental replicates.

1.2 Bayesian parameter estimation and prior probabilities

Here we use Bayesian approaches, in which not only the measurements but also the model parameters are interpreted as random variables. The objective function in this framework is the posterior distribution, which is a distribution over model parameters conditioned on the data and, according to Bayes' Theorem, given by

$$P(\theta|\mathcal{D}) = \frac{P(\mathcal{D}|\theta)P(\theta)}{P(\mathcal{D})},$$

with prior probability $p(\theta)$ that reflects prior knowledge about the parameters before having seen the data. The model evidence, also called marginal distribution, $P(\mathcal{D}) = \int P(\mathcal{D}|\theta)P(\theta)d\theta$, is obtained via marginalization over parameters and only needed for model selection. In order to cover several orders of magnitude, we log-transformed all parameters, i.e. $\theta_i = 10^{\theta_i}$, and reformulated our models accordingly. The prior distribution $p(\theta)$ includes boundaries θ_i^{lb} and θ_i^{ub} for model parameters as well as boundaries $\bar{x}^{lb}$ and $\bar{x}^{ub}$ for steady state values for the endogenous system, which cannot directly be expressed in terms of parameters:

$$p(\theta) = \begin{cases} c \cdot H(\theta) & \text{for } \theta_i \in [\theta_i^{lb}, \theta_i^{ub}] \\ 0 & \text{else} \end{cases} \tag{1}$$

$$\text{with } H(\theta) = \prod_{i=1}^{n_x} \left(\frac{(\bar{x}_i(\theta))^6}{(\bar{x}_i(\theta))^6 + (\bar{x}^{lb})^6} - \frac{(\bar{x}_i(\theta))^6}{(\bar{x}_i(\theta))^6 + (\bar{x}^{ub})^6} \right) \tag{2}$$

Here, c denotes a constant and $H(x)$ is a combined Hill function. Boundary values for steady state concentrations were set to $\bar{x}^{lb} = 10^3$ molecules per cell and $\bar{x}^{ub} = 10^9$ molecules per cell. These boundaries cover a wide range of concentrations and thus do not impose a real restriction. Furthermore, the upper bound seems plausible taking into account recent estimates of the entire proteome of HEK293 cells of about 10^{11} molecules per cell [1].

Boundaries for the parameters were selected in an iterative procedure after initial optimization runs to initialize the Markov chains for the sampling procedure, see subsequent sections for further details.

The posterior distribution is investigated via generation of representative samples $\{\theta^s : s = 1, \ldots, N\}$, for which we use Markov Chain Monte Carlo (MCMC) methods.

1.3 Modeling PKD and CERT interactions at the trans-Golgi network of mammalian cells

The two model variants A and B are given in Table S1. The first column describes the structure of the ODE model, equations for fluxes are listed in the second column, and output variables are explained in the third column. Variables x_1 to x_4 refer to PKD and PI4KIIIβ. Corresponding equations are the same for both models except from the flux V_{11}, which describes CERT-mediated activation of PKD via ceramide transport and conversion into DAG. In model A this flow depends on the rate of CERT recruitment to the TGN via PI4P (V_{31}), while in model B it depends on the fraction of unphosphorylated CERT that is bound to both membranes (x_7). CERT is described by variables x_5 to x_7, and respective equations differ for both model variants: In model A a positive feedback between CERT dependent ceramide transfer (increasing with V_{31}) and the PKD activation rate V_{11} is formed by the overall reaction structure. In model B a negative feedback between CERT dependent ceramide transfer (increasing with x_7) and the PKD activation rate V_{11} is formed by the overall reaction structure. We used the following two kinetics for reaction rates:

1. Basal synthesis rates and synthesis rates induced by ectopic expression were described by constant reaction rates S.

2. Linear kinetics
$$f_1(x_a, \tilde{\theta}_1) = \tilde{\theta}_1 x_a$$
was used for degradation rates, basal activation of PKD and activation via PKD activators and dephosphorylation of CERT at the ER.

3. Regulatory influences across different key proteins of the model were described by Michaelis Menten type kinetics,
$$f_2(x_a, x_b, \tilde{\theta}_1, \tilde{\theta}_2) = \tilde{\theta}_1 x_a \frac{x_b}{x_b + \tilde{\theta}_2}$$
to reflect that the effect of species x_b on the flux is bounded from above for x_b values far above the threshold $\tilde{\theta}_2$. This includes PI4KIIIβ activation via active PKD (V_{22} in both models), PI4KIIIβ dependent recruitment of CERT to the TGN (V_{31} in model A, V_{33} in model B) and detachment of

CERT from the TGN upon phosphorylation via PKD (V_{33} in model A, V_{34} in model B). Activation of PKD by CERT dependent ceramide transfer (V_{11} in both models) is also modeled by a f_2 function.

Experimental perturbations are described by input parameters u, which are switched from 0 to 1 when the perturbation is active. The respective manipulation then enters the model via a term $p_u u$ with parameter p_u that is included in the parameter vector θ. Integrating p_u into the parameter vector allows for input uncertainty which is very important for calibrating regulatory models (see e.g. [2]). We note here that this is a simple description of an external input, which serves the purpose to estimate the effect of a particular manipulation such as a specific dose of vector construct added to the cell culture. If the model is to be used to predict the behavior for different treatments, this probably requires the inclusion of a more advanced input model.

Since every model variable has its individual degradation rate constant, mass is not conserved in our models. In the long term the system reaches a dynamic equilibrium with constant concentrations and reaction fluxes.

2 Absolute quantification of proteins

Adding estimates of absolute protein abundances to the dataset does improve the model, enabling the prediction of absolute abundances in different scenarios after calibration. Commercially available standards to generate a calibration curve were only available for PI4KIIIβ. We used this standard to validate our own experimental procedure for PI4KIIIβ quantification. Then we used this procedure to estimate absolute abundances of PKD and CERT as well. The method can be divided into several steps, each comprising wet lab procedures and error analysis and is explained in the following.

2.1 Linear regression using a trans-blot GFP standard

First we established a GFP-protein standard with a commercially available pre-quantified GFP-protein solution. We prepared four replicates of seven differing GFP-protein dilutions and blot them using a Western Blot gel, as depicted in Figure 2A in the main article. The gels are prepared, incubated with IR-antibodies and subsequently quantified according to the wet lab and image quantification section. The measured signals and the known amount of GFP-protein (in molecules/lane) serve to establish a trans-blot standard curve. We calibrated a standard linear regression model

$$\mu_{\mathrm{SPL}} = a\mu_{\mathrm{MPL}} + b \tag{3}$$

with molecule number per lane μ_{MPL}, GFP-signal per lane μ_{SPL} and estimates of the regression parameters a and b. Maximum likelihood estimates of these parameters along with their uncertainties, as well as model fit statistics, are directly obtained from the MATLAB2011b linear regression tools, see values in Figure 2A. Since we used multiple gels for the generation of the standard, we were able to use 28 (4×7) replicates to calibrate our regression model.

2.2 Estimation of ectopic expression abundances

Once the trans-blot GFP standard was established, it was used to quantify GFP-tagged proteins in samples of cell lysates from ectopic expression experiments. We performed a cell culture experiment and ectopically expressed in three wells a GFP-tagged version of the target protein GFP-PI4KIIIβ in HEK293T cells.

We additionally grew three untreated cell cultures, determined the culture confluence and harvested all six cultures after 24 h. Next we blotted three lysates from three wells with ectopically expressing cells and detected with GFP antibodies. Image data files of the Western Blots are available as online material. Then we used the regression model (equation (3)) to inversely predict the mean expressed GFP-tagged target protein amount in the lanes (results in Figure 2A):

$$\mu^i_{\mathrm{OML}} = \frac{\mu^i_{\mathrm{OSL}} - b}{a}. \tag{4}$$

In this case μ^i_{OML} is the molecule number per lane in the ectopic expression case and μ^i_{OSL} is the ectopic expression signal per lane.

For estimation of the uncertainty of this prediction we use the Delta Method [3],

$$\sigma^i_{\text{OML}} = \frac{\sqrt{\frac{SS_{\text{res.y}}}{N-2}}}{\hat{a}} \cdot \sqrt{1 + \frac{1}{N} + \frac{(\mu^i_{\text{OML}} - \bar{x}_{\text{std}})^2}{\sum_{i=1}^{N}(x_i - \bar{x}_{\text{std}})^2}}. \tag{5}$$

It employs the sum of squares of the y-residuals $SS_{\text{res.y}}$ of the regression, the number of replicates N to establish the regression model, the mean $\bar{x}_{\text{std}}$ of x-values x_i and the regression model estimate of ectopically expressed molecules per lane μ^i_{OML} (see equation (4)). Since WB signals of these samples were generated with different loads, which is a standard procedure to screen for load artifacts, we first normalized all predicted lane means and standard errors to the loads: $\mu^i_{\text{OMC}} = \mu^i_{\text{OML}}/N^i_{\text{cells}}$ and $\sigma^i_{\text{OMC}} = \sigma^i_{\text{OML}}/N^i_{\text{cells}}$. Here μ^i_{OMC} are the average ectopically expressed molecules per cell and σ^i_{OMC} the respective standard errors. Theoretically the means should now be the same but measurement noise and the regression model prediction uncertainty requires a weighted estimate of the mean and standard error at this step:

$$\hat{\mu}_{\text{OMC}} = \frac{\sum_{i=1}^{N} \sigma^i_{\text{OMC}} \mu^i_{\text{OMC}}}{\sum_{i=1}^{N} \sigma^i_{\text{OMC}}}, \tag{6}$$

$$\hat{\sigma}_{\text{OMC}} = \sqrt{\frac{\sum_{i=1}^{N} \sigma^i_{\text{OMC}} (\mu^i_{\text{OMC}} - \mu_{\text{OMC}})^2}{\sum_{i=1}^{N} \sigma^i_{\text{OMC}}}}. \tag{7}$$

We now have estimates of the average protein abundance per cell $\hat{\mu}_{\text{OMC}}$ and the standard error $\hat{\sigma}_{\text{OMC}}$.

2.3 Estimation of endogenous abundances

In the next step the endogenous protein abundance was determined from the protein abundances in the ectopically expressing cells. Therefore we established Western Blots of three endogenous cell culture lysates together with three lysates of the ectopically expressing cells. We detected with a protein specific antibody (here: anti-PI4KIIIβ), see Figure 2B.

(**Remark:** We blotted the endogenous cell culture samples and the ectopic expressed samples that are used in the second step already on the same gel. A second gel can be omitted if a two-channel IR-detection device is used together with different secondary antibodies for anti-GFP and anti-PI4KIIIβ.)

All three ectopic expression signals y^i_{OSL} and endogenous signals y^i_{ESL} must be normalized with their respective amount of cells per lane $N^i_{\text{O.cells}}$ and $N^i_{\text{E.cells}}$, respectively,

$$y^i_{\text{ESC}} = y^i_{\text{ESL}}/N^i_{\text{E.cells}} \tag{8}$$

$$y^i_{\text{OSC}} = y^i_{\text{OSL}}/N^i_{\text{O.cells}}. \tag{9}$$

We adapted the molecule numbers in the load by altering the dilution factor in the cell lysis in the endogenous and ectopically expressed samples. This was done in order to avoid saturation effects in the signals. It turned out that a lysate dilution difference of a factor of five, i.e. 200μL endogenous cell lysate and 1000μL ectopic expression cell lysate, provides reasonable signals. Ratios y^i_{rat} between endogenous and ectopic expression signals were calculated along with the mean and standard error of these estimates:

$$y^i_{\text{rat}} = \frac{y^i_{\text{ESC}}}{y^i_{\text{OSC}}} \tag{10}$$

$$\mu_{\text{rat}}. = \frac{1}{N} \sum y^i_{\text{rat}} \tag{11}$$

$$\sigma_{\text{rat}} = \sqrt{\frac{1}{N-1} \sum y^i_{\text{rat}} - \mu_{\text{rat}}} \tag{12}$$

Finally, we used Gaussian error propagation to calculate the mean and standard error of endogenous protein abundances from the expression ratios and the ectopically expressed protein abundances:

$$\mu_{\text{EMC}} = \hat{\mu}_{\text{OMC}} \cdot \mu_{\text{rat.}} \tag{13}$$

$$\sigma_{\text{End.}} = \tag{14}$$

$$\sqrt{\left(\frac{\partial \mu_{\text{EMC}}}{\partial \mu_{\text{OMC}}}\sigma_{\text{Ov.Exp.}}\right)^2 + \left(\frac{\partial \mu_{\text{EMC}}}{\partial \mu_{\text{rat.}}}\sigma_{\text{rat.}}\right)^2}. \tag{15}$$

These estimates were finally used as experimental data in the likelihood function.

2.4 Validation

For validation we created a dilution series from a commercially available quantified GST-PI4KIIIβ protein solution (Figure 2B). This was used to quantify both endogenous and ectopically expressed protein abundance directly by using the same linear regression procedure as with the GFP-standard. Results are depicted in Figure 2C, which shows the comparison of our estimate of the amount of PI4KIIIβ and that of the commercial standard. With our method we obtain an estimate of $8.0 \cdot 10^7 \pm 1.9 \cdot 10^7$ PI4KIIIβ molecules for the ectopic expression experiment, and $1.9 \cdot 10^6 \pm 8.9 \cdot 10^5$ molecules for the endogenous amount of protein. Using the commercial standard we obtain estimates of $1.3 \cdot 10^8 \pm 4.0 \cdot 10^7$ and $9.8 \cdot 10^5 \pm 4.2 \cdot 10^5$ molecules for the ectopic expression experiment and the amount of endogenous protein, respectively. Thus the means of both estimates differ by a factor of less than two, and in particular, they agree in the order of magnitude. Due to the usual measurement noise of Western blotting methods and conservative error tracking, the final coefficients of variation varied between 25% and 40% in both methods. We conclude that our approach provides reliable first estimates for the absolute abundance of PI4KIIIβ, and, based on these results, we decided to apply the procdure for estimation of PKD and CERT amounts as well.

2.5 Quantification of PKD and CERT

Image data and spreadsheet calculations for the same quantification procedure applied to PKD and CERT are available as online materials.

Since we decided during the project to use FLAG-CERT expression constructs for the time series data instead of GFP-CERT, we used the same procedure to estimate the ectopic expression ratios of FLAG-CERT and calculated the ectopically expressed amount from the endogenous estimates from the GFP-CERT calculations. To account for the three biological replicates that led to these estimates we included the mean estimates of all absolute quantifications as three identical data points in the final dataset.

3 Time series data

We established the time series experiments that were finally used for model calibration in three steps. First initial snapshot experiments were performed to assess which perturbations imply a significant excitement of the system. These experiments were repeated in a second step with an increased time resolution. Finally, specific refinement experiments were designed by analyzing model predictions of uncertainties in selected scenarios.

3.1 Initial snapshot experiments

As described in the main article, we decided to bring PI4KIIIβ and CERT in an ectopically expressed state to increase signal strength before applying further perturbations. The results of the snapshot experiments are depicted in Figure S1.

In the Western Blot experiments of Figure S1A we expressed GFP-PI4KIIIβ, in Figure S1B we expressed GFP-CERT, both for 24 h. The first columns of A and B feature no additional perturbation. The ectopic expression already caused an excitation of the system, which is visible in the PKD signal levels in the first columns. It can be seen that the active PKD signal was higher when CERT was ectopically

expressed (B) in comparison to PI4KIIIβ (A). Subsequently, 24 h after initiating the ectopic expression we perturbed PKD activity via the PKD activator PDBu and the PKD inhibitor kb-NB142-70, respectively. In both Western Blots the second and third columns depict $0.25h$ treatment with PKD activator and $1h$ treatment with inhibitor, respectively. In the fourth columns combined treatments of 1h inhibitor and $0.25h$ activator are shown. Therefore phospho-PKD signals increase in the second column, decrease in the third column and have intermediate levels in columns four. Active PI4KIIIβ shows the same qualitative behavior as active PKD. In the second column of Figure S1A the highest increase in signal was observed for active PKD and PI4KIIIβ 15 minutes after PDBu stimulation. In Blot B similar qualitative changes were observed for phospho-PKD signals but with an overall stronger signal. A drop of both phospho-PKD and phospho-CERT signal intensity was observed in the third column of Figure S1B after one hour of PKD inhibition with kb-NB142-70. We decided to include the PI4KIIIβ expression with activator and CERT expression with inhibitor into our main time series, since these experiments resulted in visible qualitative changes for both measured quantities.

3.2 Time series experiments

Figure 3 of the main article shows Western Blots of representative replicates for time series of the two selected perturbation experiments. These experiments were chosen from the set of snapshot experiments in Figure S1, but with a higher time resolution. To avoid cross detection as it was occasionally observed with PKD, we switched to a FLAG-CERT construct for the vector expression that runs on a different molecular weight. 24 h after ectopically expressing PI4KIIIβ we added PDBu and measured 0, 5, 10, 15, 30 and 60 min after the perturbation. In the second perturbation experiment, kb-NB142-70 was added 24 h after ectopically expressing CERT and measurements were taken 0, 15, 30, 60, 120 and 180 min after the perturbation.

3.3 Data normalization

We quantified the phosphorylation state of the proteins to assess the dynamic response of the system. Two normalizations were applied to all phospho-specific-signals, and respective model outputs were adapted accordingly. For PKD we first normalized signals to tubulin to account for loading differences, leading to a signal that is proportional to the amount of phospho-PKD

$$z_{\text{PKD,norm.tub.}} \propto x_{\text{phospho. PKD.}} \tag{16}$$

with $z_{\text{PKD,norm.tub.}}$ being the signal normalized to tubulin and $x_{\text{phopho. PKD.}}$ being the amount of phospho-PKD in the cell.

For PI4KIIIβ and CERT we used a different normalization. In ectopic expression experiments the overall abundance was dominated by the artificially introduced tagged protein (see results 'Absolute quantification'), whose abundance was several orders of magnitude higher than the natural protein. Hence the GFP or FLAG tag-signal can be assumed proportional to the overall abundance. For this reason, in the case of phospho-CERT and phospho-PI4KIIIβ, which are exclusively studied in ectopically expressed states, we first normalized to anti-FLAG or anti-GFP signals, respectively. A normalization to the tag-signal returned a signal proportional to the relative phosphorylation proportion of these proteins

$$z_{\text{PI4KIII}\beta\text{ norm.tag.}} \propto x_{\text{PI4KIII}\beta\text{ phospho. prot.}}/x_{\text{PI4KIII}\beta\text{ total. prot.}} \tag{17}$$

$$z_{\text{CERT norm.tag.}} \propto x_{\text{CERT phospho. prot.}}/x_{\text{CERT total. prot.}}, \tag{18}$$

with $z_{\text{CERT norm.tag.}}$ being the signal normed to the tag, $x_{\text{CERT phospho. prot.}}$ being the amount of phospho-protein and $x_{\text{total. prot.}}$ being the amount of total protein (respectively for PI4KIIIβ).

In a second step all signals were additionally normalized to a specific time point by using the values $z_{\text{PKD,norm.tub.}}$, $z_{\text{CERT norm.tag.}}$ and $z_{\text{PI4KIII}\beta\text{ norm.tag.}}$ from the first normalization.

Combining these two normalization steps, the result for e.g. the fraction of phosphorylated CERT

reads

$$z_{\mathrm{CERTpRN24}}(t) = \frac{z_{\mathrm{CERT\ norm.tag.}}(t)}{z_{\mathrm{CERT\ norm.tag.}}(t=24h)} = \frac{x_{\mathrm{CERT\ phospho.\ prot.}}(t)/x_{\mathrm{CERT\ total.\ prot.}}(t)}{x_{\mathrm{CERT\ phospho.\ prot.}}(t=24h)/x_{\mathrm{CERT\ total.\ prot.}}(t=24h)}$$

In this case $z_{\mathrm{CERTpRN24}}(t)$ is the normalized data that was finally used to calibrate the model. The respective model outputs were normalized accordingly:

$$y_7 = y_{\mathrm{CERTpRN24}} = \frac{x_6(t)/(x_5(t)+x_6(t)+x_7(t))}{x_6(t=24h)/(x_5(t=24h)+x_6(t=24h)+x_7(t=24h))}.$$

The variables x_5, x_6 and x_7 represent the different CERT variants in the model (see Table S1). The data point at the time that was used for normalization, here $t = 24h$, was consistently removed from the dataset.

3.4 Time series refinements via model based experimental design

Figure S6D and Figure 4D show results of refinement experiments that were proposed by a Bayesian approach for experiment design which proposes experiments that minimize expected variances (see [4] for further details).

4 Markov Chain Monte Carlo (MCMC) sampling

The parameters to be estimated from experimental data include synthesis and degradation rate constants s and a, respectively, reaction rate constants p for conversions, threshold values m and experimental input parameters p_u. Model A comprises 26 parameters, model B has 27 parameters.

4.1 Sampling strategy

We implemented a parallel tempering population MCMC algorithm similar to Calderhead *et. al.* [5], with an adaptive Metropolis kernel and acceptance rate dependent scaling for each temperature, which enables fast mixing. The shape of the kernel for each temperature was individually determined by the covariance matrix of the sample. Temperature swaps are proposed after each standard Metropolis update step. The scaling of the covariance matrix was initially set to 1 and was subsequently increased by 10% if the acceptance rate exceeded 95% or decreased by 10% in case the acceptance rate was lower than 5%. Temperature scaling was chosen according to a power series of five, i.e.

$$T_n = \left(\frac{n}{10}\right)^5$$

$$P(\theta|\mathcal{D})^n = \frac{P(\mathcal{D}|\theta)^{T_n} P(\theta)}{P(\mathcal{D})^n}$$

with temperature T_n, power posterior $P(\theta|\mathcal{D})^n$, power likelihood $P(\mathcal{D}|\theta)^{T_n}$, prior $P(\theta)$, and temperature specific evidence $P(\mathcal{D})^n$. In this setting T_{10} corresponds to the posterior and T_1 represents the highest temperature. Figure S2 shows the projection of the sample from a MCMC run of model A onto a two-dimensional subspace from the posterior T_{10} and a higher temperature T_8.

Scaling of transition kernels, within chain acceptance rates, and temperature swapping acceptance rates are depicted in Figure S3 for a representative run. It is clearly visible that a good acceptance rate between 20 and 50 percent is achieved between all neighboring temperature pairs, which confirms that the initially chosen power series was an appropriate choice. As expected, scaling of the kernel decreases for lower temperatures. The acceptance rate within a chain is between 10 and 20 percent, which we consider a good value for an adaptive Metropolis scheme with 27 dimensions and additional constraints concerning the steady states. Kernel scalings decrease from high temperatures towards the posterior due to additional rejections originating from the increasing influence of the likelihood function.

4.2 Initial optimization and parameter constraints

To initialize MCMC sampling we conducted initial optimization of the likelihood function. Therefore we used the interior point optimization routine implemented in MATLAB's local optimization routine 'fmincon' with an objective function tolerance of 10^{-6} and uniformly sampled initial values. The boundaries that we used for this purpose are listed in Table S2.

Boundaries for parameters with linear influence on reaction rates allow for a variation of these parameters between ten orders of magnitude, which we consider an adequately large range of values. Boundaries for degradation rates result from protein half lifes between about 30 seconds and nine months, in agreement with published values [6]. Synthesis rates range from several hundred up to ten million proteins per hour, which we believe to be a conservative upper bound for the capabilities of the cellular protein production machinery. Input parameters $\tilde{p}_u$ have different meanings in our modeling framework such as for example scaling factors for production rates or amplification of reactions rates. We do not allow them to be smaller than 10^{-1}, which we consider a failed experiment. Their upper limit is motivated by the case when input scalings define artificial production rates or system inflows, which we do not allow to be more than ten million molecules per hour (for the same reasons we limit the natural production rates in this way). Boundaries of threshold parameters $\tilde{m}$ are allowed to be one order of magnitude higher/lower than the allowed ranges of steady states (see equation (2)) to provide enough flexibility that $\tilde{m}$ can act as saturation threshold over the entire range of permitted steady state values.

The twenty best solutions from 1000 optimization runs with different initializations were used to choose appropriate boundaries for a $\log_{10}$-uniform prior distribution $P(\theta)$ for subsequent MCMC sampling. For this we subtracted and added one order of magnitude to the lowest and highest values of these solutions, respectively, which we consider a rather conservative approach.

4.3 Evidence and Bayes factor

Calculation of the Bayes factor for model comparison requires the calculation of the marginal likelihoods for each model,

$$P(\mathcal{D}) = E_{P(\theta)}[P(\mathcal{D}|\theta)] = \int P(\mathcal{D}|\theta)P(\theta)d\theta. \tag{19}$$

This was done by implementing the thermodynamic integration scheme

$$\log P(\mathcal{D}) \approx \frac{1}{2}\Delta t_n \sum_n (E_{n-1} + E_n)$$

described in Calderhead *et. al.* [5], with Δt_n being the temperature difference between two neighboring temperatures t_n and t_{n-1}. Expectation values E_n and E_{n-1} are defined as

$$E_n = E_{\theta|\mathcal{D},t_n}[\log P(\mathcal{D}|\theta)] \approx \frac{1}{N_n}\sum_i \Delta t_n \log P(\mathcal{D}|\theta_i)$$

$$E_{n-1} = E_{\theta|\mathcal{D},t_{n-1}}[\log P(\mathcal{D}|\theta)] \approx \frac{1}{N_{n-1}}\sum_i \Delta t_n \log P(\mathcal{D}|\theta_i).$$

with sample sizes N_n and N_{n-1}, respectively.

Here, $E_{\theta|\mathcal{D},t_n}$ and $E_{\theta|\mathcal{D},t_{n-1}}$ denote expectation values with respect to the power posteriors with temperatures t_n and t_{n-1}, respectively. MCMC samples from these power posteriors are good choices for support points that can be used for Monte Carlo integration required in Equation (19). From the marginal likelihoods $P_{M_A}(\mathcal{D})$ and $P_{M_B}(\mathcal{D})$ of both models we computed the log-Bayes factor according to

$$2\log K_{A,B} = 2(\log P_{M_A}(\mathcal{D}) - \log P_{M_B}(\mathcal{D}))$$

These values can directly be compared to published evidence tables [7].

4.4 MCMC convergence and reproducibility of numerical results

Parallel tempering MCMC runs for both models have been repeated three times. Chain starting values have been sampled from the ten best results of the initial optimization runs. Mean and standard error for the Bayes factor were empirically estimated using the results from three independent runs. Convergence of the Markov Chains was tested via the Gelman Rubin statistics [8], which is suited for parallel chains. The test routine was applied individually for each temperature. All estimates for the $\hat{R}$ values have been below 1.1, indicating a negative test result for non-convergence. Results of the convergence testing are depicted in Figure S4.

5 Implementation details of Bayesian analysis

The complete Bayesian model analysis is implemented as a MATLAB R2011b (64 bit) script, which is structured as follows for each of the models:

1. **Initialize:** Load the settings, the SBmodel (∗.txt) and the preprocessed data (∗.xls) as MATLAB objects/variables. Define initial boundaries for all parameters.
2. **Optimization**: Run 1000 local minimizations using the MATLAB fmincon function. Initial values for the user supplied objective function are sampled log_{10}-uniformly within the initial boundaries.
3. **Initial assessment:** Visually inspect the initial model fits by comparing trajectories using the optimal parameters together with the experimental data.
4. **MCMC sampling**: Use a bounded $\log_{10}$-uniform prior for all parameters by adding/subtracting one order of magnitude from the minimum/maximum values of the best 10 initial fits. Sample initial values for the MCMC sampling from the set of the best initial model fits. Draw 500k samples from the posterior distribution using a ten temperatures parallel population MCMC algorithm. This sampling procedure is repeated three times.
5. **Convergence analysis**: The chains are tested for convergence for each temperature using the Gelman Rubin statistics. All parameters have to fulfill $\hat{R} < 1.1$ for all temperatures to pass the test.
6. **Predictions**: 5000 parameter vectors are drawn equally spaced from the parameter sample, which are used to simulate trajectories for different scenarios. Reconstruction of probability densities for relevant quantities are calculated via standard kernel density estimates. For example, the time evolution of the density of states is determined via density estimates with these simulated trajectories for a discrete grid on the time axis.
7. **Model comparison**: The marginal likelihood is calculated using thermodynamic integration, using a representative subsample of 5000 sample points. The three replicates of the MCMC chains are used to estimate the variance. Bayes factors are calculated from the marginal values of both models and equal prior probabilities.

6 Further supporting results

6.1 Model comparison

Marginal likelihoods for models A and B and the Bayes factor are shown in Figure S5. Standard deviations for the marginal distributions are estimated from three independent sampling runs. For estimation of the error of the Bayes factor we employed Gaussian error propagation. The result ($2 \log K_{A,B} = 12.3 \pm 0.4$) indicates very strong evidence for model A according to [7].

6.2 Data fit model B

Figure S6 displays the Bayesian model fit for model B (compare Figure 4 for model A), without the validation sub-figure 'E'.

Misfit of the experimental data of model B are visible in the refinement measurements, shown in the left plot of Figure S6D. It can be seen that the model structure cannot capture the fast increase of PKD activity 3 and 6 hours after ectopic expression of PI4KIIIβ. Steady state simulations in Figure S6A are slightly less perfect compared to model A. Taken together these differences result in a Bayes factor that describes model A superior to model B (evidence: 'very strong').

Although the Bayes factor ranks model A above model B, there are still parts of the data that can be reproduced by model B. The response of the system in the first experiment, ectopic expression of PI4KIIIβ for 24h followed by PKD activation, is comparably well captured by model B (Figure S6B). Similar results hold for the second experiment, in which the response to ectopic expression of CERT and inhibition of PKD after 24h was measured (Figure S6C).

Figures

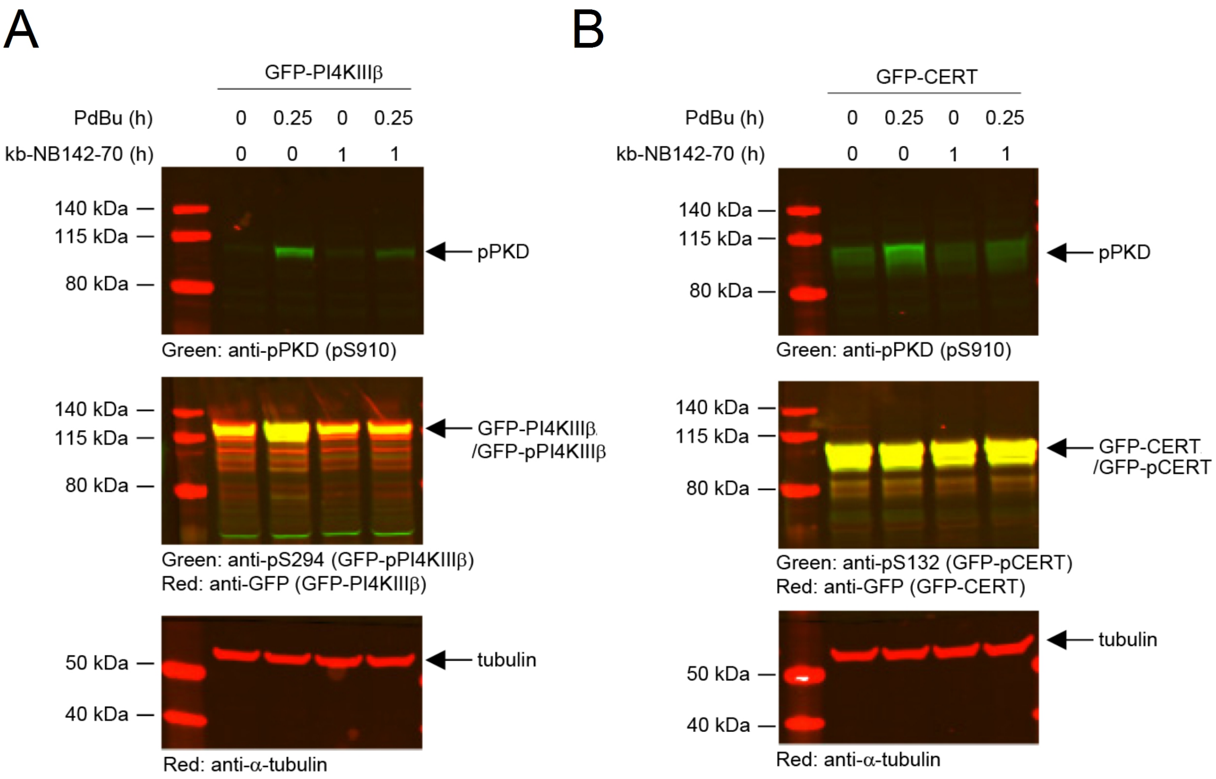

Figure S 1. Western Blots of initial snapshot experiments. (A) HEK293T cell cultures do ectopically express GFP-PI4KIIIβ for 24h. (B) HEK293T cell cultures do ectopically express GFP-CERT for 24h. Subsequent single and combined treatments of the supernatant with PKD activator PDBu and PKD inhibitor kb-NB142-70 were performed. Phosphospecific antibodies for PKD, PI4KIIIβ and CERT monitor changes in the activation status of the proteins (green channel). Overall abundance of GFP-PI4KIIIβ and GFP-CERT was monitored with GFP-antibodies (red channel).

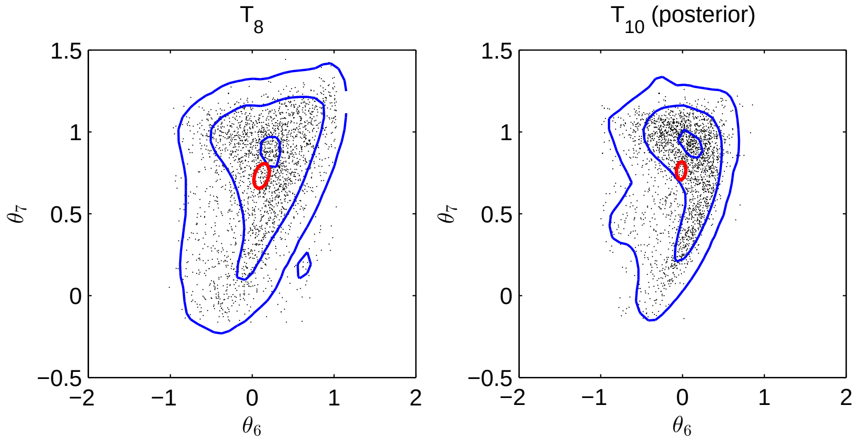

Figure S 2. Parallel tempering MCMC illustration. Two-parameter plot of samples from the MCMC run of model A from the posterior T_{10} and a higher temperature T_8. Samples are represented by black dots, blue lines are level sets obtained via an estimate of the marginal density, and red ellipsoids represent the shape of the transition kernel.

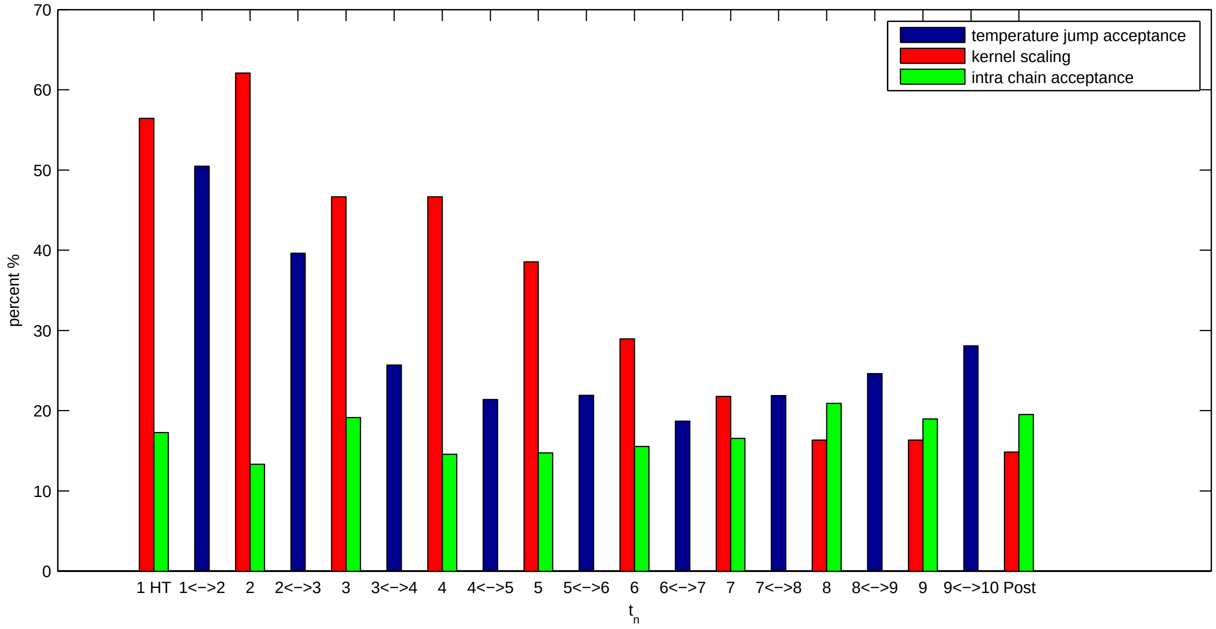

Figure S 3. Acceptance rate statistics of a representative run. Shown are the intra-chain aceptance rate and kernel scaling for each temperature, and temperature jump acceptance rates between temperatures.

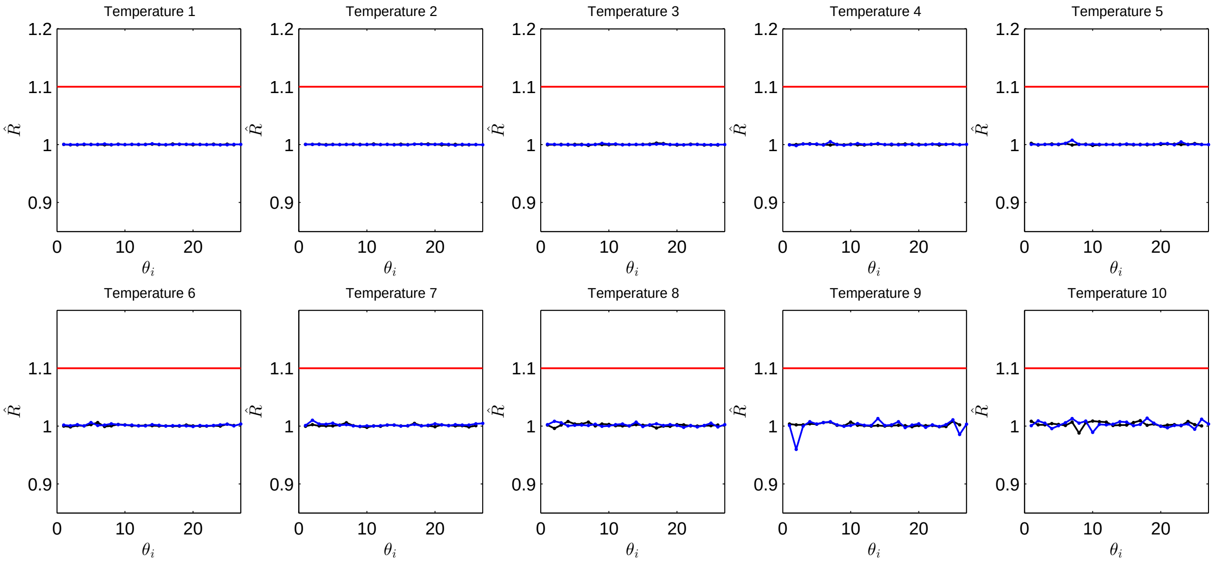

Figure S 4. MCMC convergence results. For all temperatures three population MCMC runs with different starting values were performed. Chains were tested according to the Gelman Rubin statistics. Black and blue lines denote results for Model A and B, respectively. All parameters are below the recommended value $\hat{R} \leq 1.1$ for each temperature.

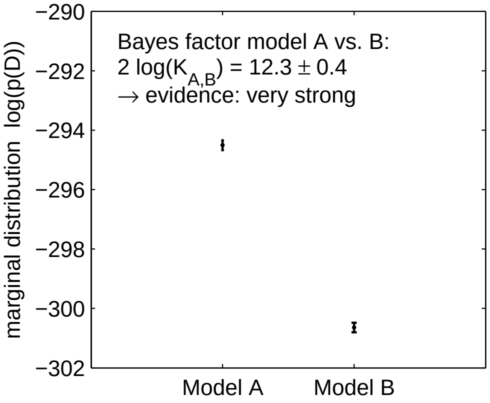

Figure S 5. Model comparison via Bayes factors. Depicted are the log-marginal likelihoods for both models and the resulting log-Bayes factor. Standard deviations are estimated from three independent sampling runs for both models.

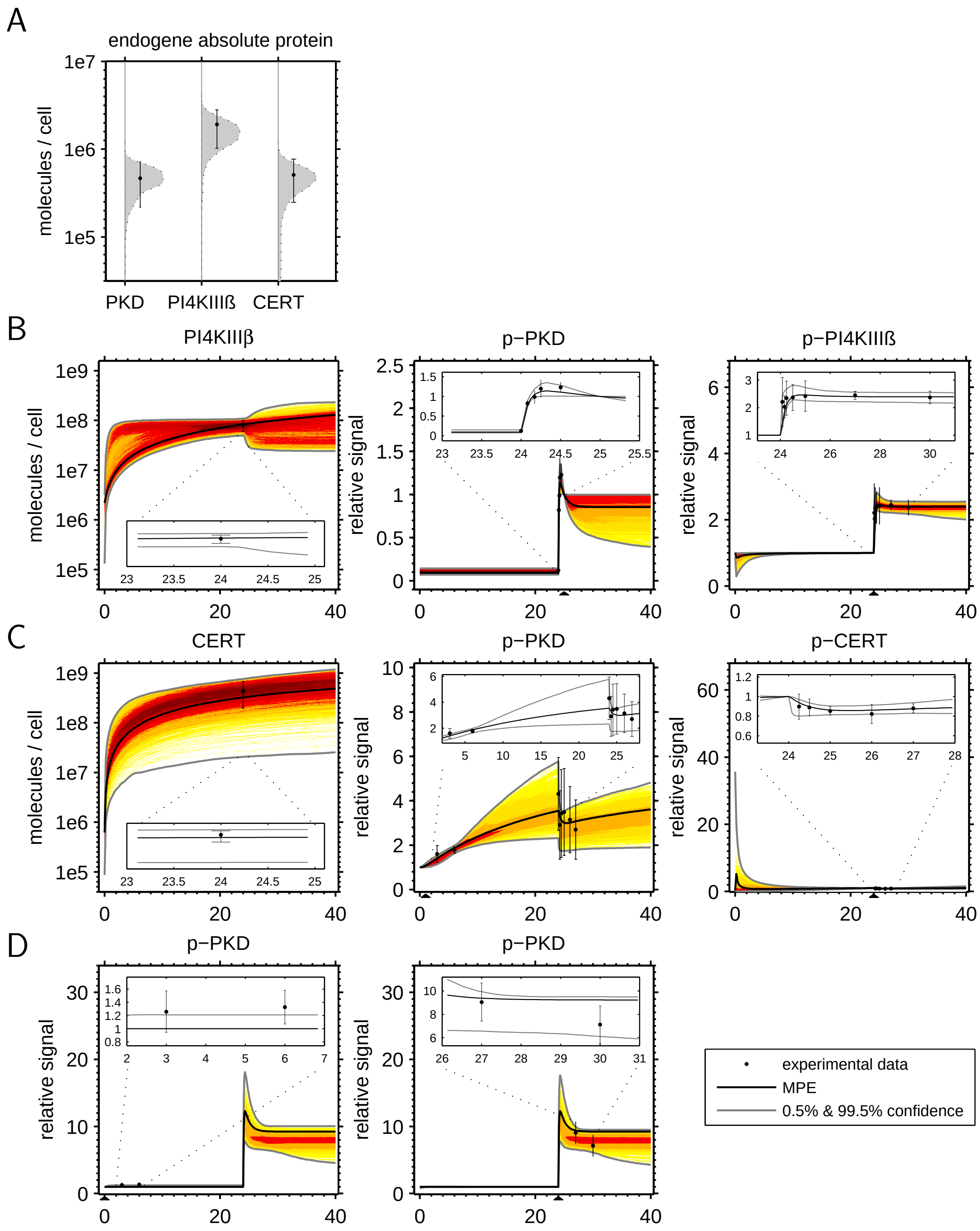

Figure S 6. Data fit model B. (A) Distributions for the endogenous steady states of PKD, PI4KIIIβ and CERT, together with experimental results from absolute quantification experiments. (B-D) Dynamic responses to system perturbations in terms of color encoded, time dependent state densities that were estimated on a discrete grid on the time axis from representative posterior trajectories. Black and grey lines correspond to the Maximum posterior estimate and 99.5% confidence intervals, respectively. (B) Ectopic expression of PI4KIIIβ and subsequent activation of PKD via PDBu at $24h$. Depicted are the overall PI4KIIIβ level and the phosphorylation status of PKD and PI4KIIIβ. (C) Ectopic expression of CERT with subsequent inhibition of PKD via kb-NB142-70 after $24h$. Shown are the overall CERT level and the phosphorylation status of PKD and CERT. (D) Refinement measurements of early stage and long-term PKD activity measured when repeating the experiment from B.

Tables

ODE structure	Reaction rate equations	Output variables
Subsystem 1: PKD<br>$\dot{x}_1 = -V_{11} - V_{12} + V_{13} + S_{12} + S_{13} - D_{13}$<br>$\dot{x}_2 = +V_{11} + V_{12} - V_{13} - D_{14}$<br>Subsystem 2: PI4KIII$\beta$<br>$\dot{x}_3 = +V_{21} - V_{22} + S_{21} + S_{22} - D_{21}$<br>$\dot{x}_4 = -V_{21} + V_{22} - D_{22}$<br><br>Variable names in the model files:<br>$x_1 \hat{=}$ PKD<br>$x_2 \hat{=}$ PKDpDAG<br>$x_3 \hat{=}$ PI4K3B<br>$x_4 \hat{=}$ PI4K3Bp	Subsystem 1: PKD<br>$V_{12} = f_1(x_1, \tilde{p}_{12})(1 + u_5 \tilde{p}_{u_5})$ → basal and experimental PKD activation<br>$V_{13} = f_1(x_2, \tilde{p}_{13})(1 + u_6 \tilde{p}_{u_6})$ → basal and experimental PKD inhibition<br>$S_{12} = \tilde{s}_{12}$ → basal PKD production rate<br>$D_{11} = f_1(x_1, \tilde{a}_{11})$ → PKD degradation rate<br>$D_{12} = f_1(x_2, \tilde{a}_{12})$ → active PKD degradation rate<br>Subsystem 2: PI4KIIIβ<br>$V_{21} = f_1(x_4, \tilde{p}_{21})$ → deactivation of PI4KIIIβ<br>$V_{22} = f_2(x_3, x_2, \tilde{p}_{22}, \tilde{m}_{22})$ → PKD mediated activation of PI4KIIIβ<br>$S_{21} = \tilde{s}_{21}$ → basal PI4KIIIβ production rate<br>$S_{22} = \tilde{p}_{u_3} u_3$ → experimental PI4KIIIβ production rate<br>$D_{21} = f_1(x_3, \tilde{a}_{21})$ → PI4KIIIβ degradation rate<br>$D_{22} = f_1(x_4, \tilde{a}_{22})$ → active PI4KIIIβ degradation rate	$y_1 = \frac{x_2(t)}{x_2(t = 0h)}$<br>$\hat{=}$ yPKDpN0<br>$y_2 = \frac{x_2(t)}{x_2(t = 24h)}$<br>$\hat{=}$ yPKDpN24<br>$y_3 = \frac{x_2(t)}{x_2(t = 25h)}$<br>$\hat{=}$ yPKDpN25<br>$y_4 = \frac{x_4(t)/y_7(t)}{x_4(t = 24h)/y_7(t = 24h)}$<br>$\hat{=}$ yPI4K3BpRN24<br>$y_5 = x_1 + x_2 \hat{=}$ yPKDt<br>$y_6 = x_3 + x_4 \hat{=}$ yPI4K3Bt
Subsystem 3a: Model A<br>$\dot{x}_5 = -V_{31} + V_{32} + S_{31} + S_{32} - D_{31}$<br>$\dot{x}_6 = -V_{32} + V_{33} - D_{32}$<br>$\dot{x}_7 = +V_{31} - V_{33} - D_{33}$<br><br>Variable names in the model files:<br>$x_5 \hat{=}$ CERTaER<br>$x_6 \hat{=}$ CERTpER<br>$x_7 \hat{=}$ CERTaTGN	$V_{11} = f_2(x_1, V_{31}, \tilde{p}_{11}, \tilde{m}_{11})$ → PKD activation via CERT<br>$V_{31} = f_2(x_5, x_4, \tilde{p}_{31}, \tilde{m}_{31})$ → PI4KIIIβ dependent recruitment of CERT to TGN<br>$V_{32} = f_1(x_6, \tilde{p}_{32})$ → dephosphorylation of CERT at the ER<br>$V_{33} = f_2(x_7, x_2, \tilde{p}_{33}, \tilde{m}_{33})$ → phosphorylation of CERT by PKD<br>$S_{31} = \tilde{s}_{31}$ → basal CERT production rate<br>$S_{32} = \tilde{p}_{u_4} u_4$ → experimental CERT inflow<br>$D_{31} = f_1(x_5, \tilde{a}_{31})$ → degradation of active CERT at the ER<br>$D_{32} = f_1(x_6, \tilde{a}_{32})$ → degradation of inactive CERT<br>$D_{33} = f_1(x_7, \tilde{a}_{33})$ → degradation of ER-TGN bound CERT	$y_7 = \frac{x_6(t)/y_8(t)}{x_6(t = 24h)/y_8(t = 24h)}$<br>$y_8 = x_5 + x_6 + x_7$<br><br>$y_7 \hat{=}$ yCERTpRN24<br>$y_8 \hat{=}$ yCERTt
Subsystem 3b: Model B<br>$\dot{x}_5 = -V_{31} + V_{32} + S_{31} + S_{32} - D_{31}$<br>$\dot{x}_6 = +V_{31} - V_{32} - V_{33} + V_{34} - D_{32}$<br>$\dot{x}_7 = +V_{33} - V_{34} - D_{33}$<br><br>Variable names in the model files:<br>$x_5 \hat{=}$ CERTa<br>$x_6 \hat{=}$ CERTp<br>$x_7 \hat{=}$ CERTaERTGN	$V_{11} = f_2(x_1, x_7, \tilde{p}_{11}, \tilde{m}_{31})$ → PKD activation via CERT<br>$V_{31} = f_1(x_5, \tilde{p}_{31})$ → CERT phosphorylation and binding to ER<br>$V_{32} = f_1(x_6, \tilde{p}_{32})$ → CERT dephosphorylation and detachment from the ER<br>$V_{33} = f_2(x_6, x_4, \tilde{p}_{33}, \tilde{m}_{31})$ → PI4KIIIβ triggered CERT ER binding/dephos.<br>$V_{34} = f_2(x_7, x_2, \tilde{p}_{34}, \tilde{m}_{33})$ → phosphorylation of CERT by PKD<br>$S_{31} = \tilde{s}_{31}$ → basal CERT production rate<br>$S_{32} = \tilde{p}_{u_4} u_4$ → experimental CERT inflow<br>$D_{31} = f_1(x_5, \tilde{a}_{31})$ → degradation of active CERT<br>$D_{32} = f_1(x_6, \tilde{a}_{32})$ → degradation of inactive CERT<br>$D_{33} = f_1(x_7, \tilde{a}_{33})$ → degradation of TGN-ER bound CERT	$y_7 = \frac{x_6(t)/y_8(t)}{x_6(t = 24h)/y_8(t = 24h)}$<br>$y_8 = x_5 + x_6 + x_7$<br><br>$y_7 \hat{=}$ yCERTpRN24<br>$y_8 \hat{=}$ yCERTt

Table S 1. Equations for ODE models A and B. 1. Column: Model structure and variable names in the model files, 2. Column: Reaction rate equations, 3. Column: Output variables and corresponding names according to Figure 1.

Parameter type	Boundaries
Parameters with linear influence:	$-5 \leq \tilde{p} \leq 5$
Degradation rates:	$-4 \leq \tilde{a} \leq 2$
Synthesis rates:	$2 \leq \tilde{s} \leq 7$
Input scalings:	$-1 \leq \tilde{p}_u \leq 8$
Threshold parameters:	$2 \leq \tilde{m} \leq 10$

Table S 2. Parameter boundaries for the initial optimization on a $\log_{10}$-scale.

References

1. Finka, A., Goloubinoff, P.: Proteomic data from human cell cultures refine mechanisms of chaperone-mediated protein homeostasis. Cell Stress Chaperones **18**(5), 591–605 (2013). doi:10.1007/s12192-013-0413-3

2. Kaschek, D., Timmer, J.: A variational approach to parameter estimation in ordinary differential equations. BMC Syst Biol **6**(1), 99 (2012). doi:10.1186/1752-0509-6-99

3. Parker, P.A., Vining, G.G., Wilson, S.R., Szarka, J.L., Johnson, G.: The prediction properties of classical and inverse regression for the simple linear calibration problem. J Qual Technol **42**(4), 332–347 (2010)

4. Weber, P., Kramer, A., Dingler, C., Radde, N.: Trajectory-oriented bayesian experiment design versus Fisher A-optimal design: an in depth comparison study. Bioinformatics **28**(18), 535–541 (2012). doi:10.1093/bioinformatics/bts377

5. Calderhead, B., Girolami, M.: Estimating Bayes factors via thermodynamic integration and population MCMC. Comput Stat Data Anal **53**(12), 4028–4045 (2009). doi:10.1016/j.csda.2009.07.025

6. Eden, E., Geva-Zatorsky, N., Issaeva, I., Cohen, A., Dekel, E., Danon, T., Cohen, L., Mayo, A., Alon, U.: Proteome half-life dynamics in living human cells. Science **331**(6018), 764–768 (2011). doi:10.1126/science.1199784

7. Kass, R.E., Raftery, A.E.: Bayes factors. J Am Stat Assoc **90**(430), 773–795 (1995)

8. Gelman, A., Rubin, D.B.: Inference from iterative simulation using multiple sequences. Stat Science **7**(4), 457–472 (1992)
